# Supplementary material for: Strong positive selection biases identity-by-descent-based inferences of recent demography and population structure in Plasmodium falciparum
Source: Nat Commun. 2024 Mar 20;15:2499. doi: 10.1038/s41467-024-46659-0 (PMC10954658; doi:10.1038/s41467-024-46659-0)
Supplement: Supplementary file 1 — Supplementary Information [file 41467_2024_46659_MOESM1_ESM.pdf]

Supplementary Information

Strong Positive Selection Biases Identity-By-Descent-Based Inferences of Recent Demography and Population Structure in *Plasmodium falciparum*

Guo *et al.*

Supplementary Figures

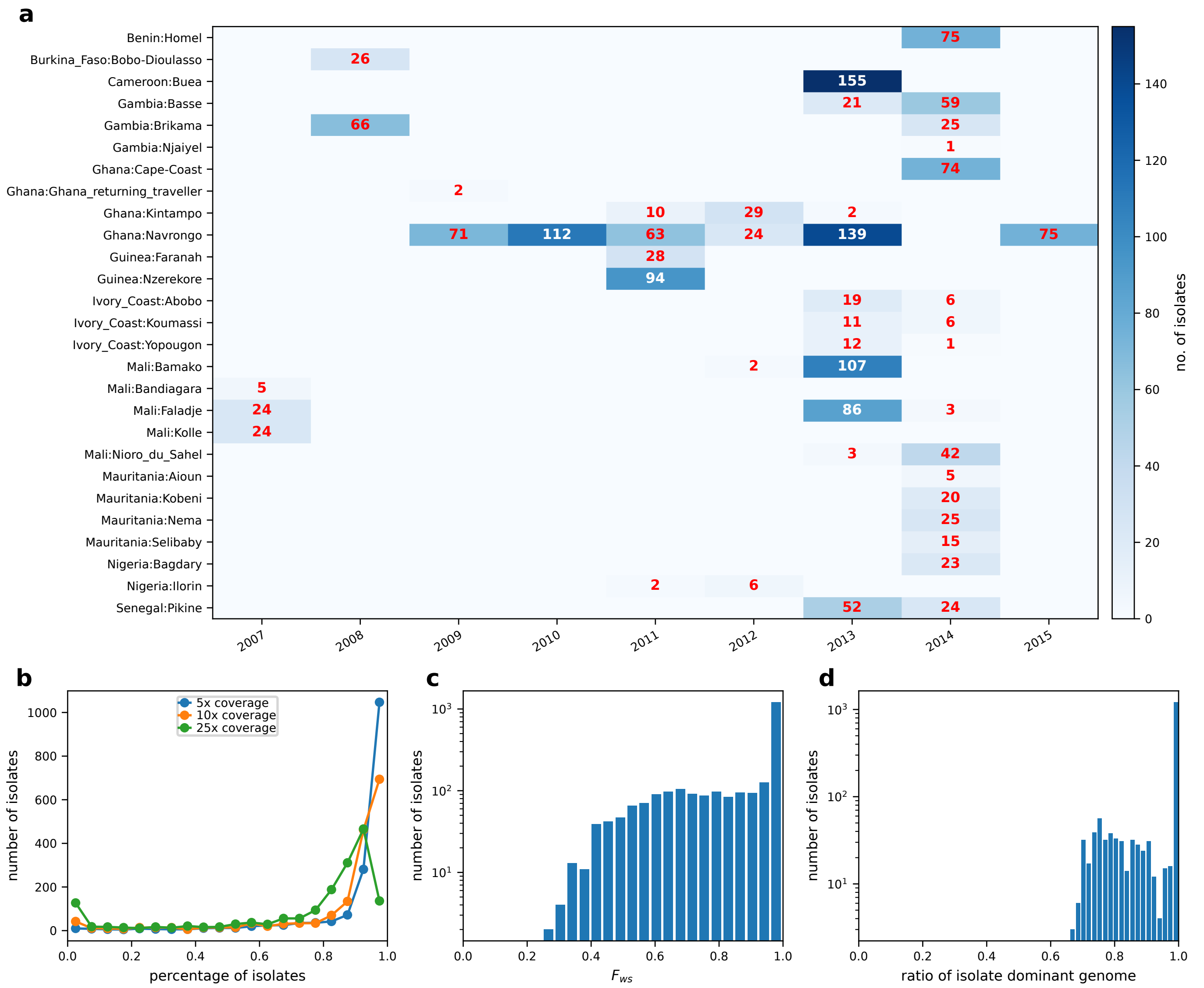

Supplementary Figure 1: Summary of *Pf* parasite isolates and WGS data from West Africa (WAF). a, Sampling location and time distribution of 1,674 analyzable samples. The text and color in each block indicate the number of isolates sampled at a given year from a given location (also see color bar). b, Distribution of genome fractions covered by at least 5, 10, and 25 sequence reads of all WGS samples from WAF. c, Distribution of F_{ws} in WGS samples that passed genotype missingness filtering. Note that to obtain more accurate distribution of F_{ws}, polyclonal isolates without a predominant clone were included in this analysis. d, Distribution of ratios of dominant genomes in in analyzable WAF isolates (determined by dEploid[1,2]). Source data are provided as a Source Data file.

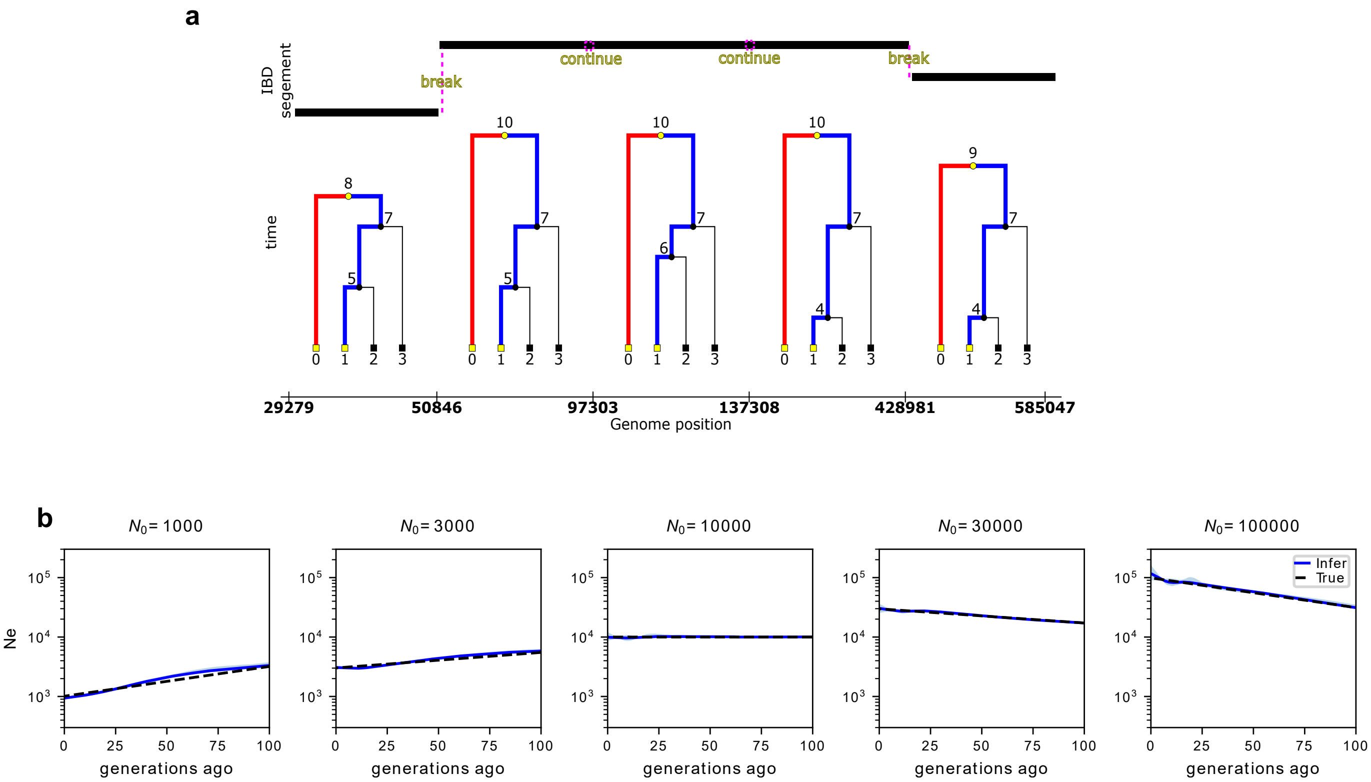

Supplementary Figure 2: True IBD inference and its validation via IBD-based N_e estimation. a, Schematic of the true IBD inference process in tskibd. For each genealogical tree along the chromosome (low panel), the most recent common ancestor (MRCA) of a pair of genomes (for instance the two with IDs 0 and 1) can be found by tracing backward (red path for genome 0 and blue path for genome 1) until the two paths meet. A theoretical IBD segment is defined by the MRCA (the origin of IBD) and span (the start and end of the IBD segment). Moving along the tree sequence, the IBD segment may break or continue at the tree junction depending on whether recombination happened to this ancestral segment (upper panel). Breaks and continuity can be determined by comparing MRCA node IDs between two nearby sampled trees. To match with most other IBD inference tools, we only include long IBD segments (such as ≥ 2 centimorgans) in the output. b, True IBD (inferred via tskibd)-based N_e estimates for the recent 100 generations are consistent with parameter population size in neutral simulations under different demographic patterns (left two: exponential decrease; middle: constant N; right two: exponential growth). Lines represent point estimates; error bands (shading areas) represent 95% confidence interval as determined by IBDNe[3]. Source data are provided as a Source Data file.

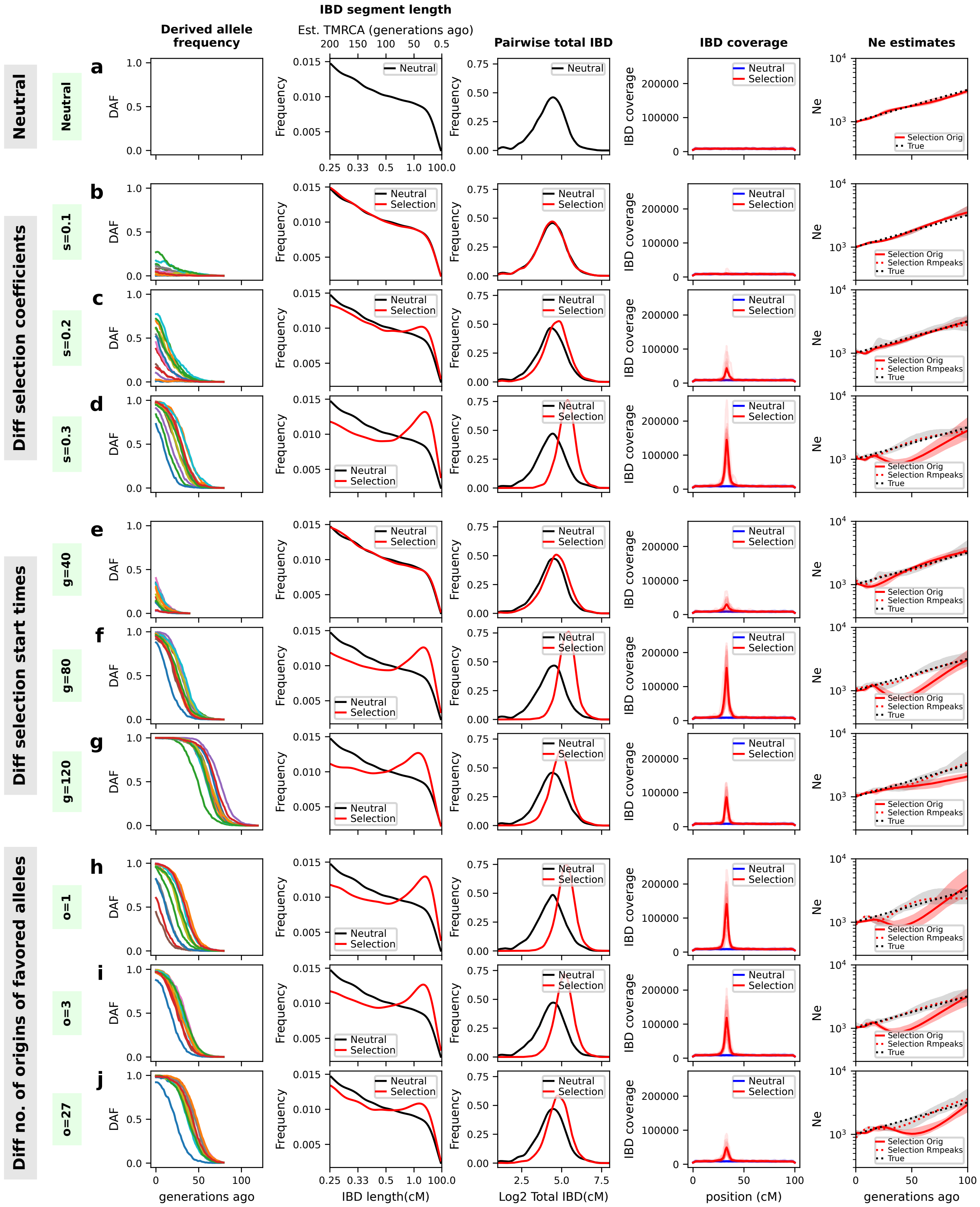

Supplementary Figure 3: Extended simulations evaluating the effect of selection on IBD distribution and N_e estimation.

Supplementary Figure 3 (cont.): a-j, Different rows represent data from different simulation cases, including neutral (a), varying selection coefficients (b-d), varying selection starting time (e-g), and varying numbers of origins of the favored allele (h-j). Column 1 includes frequency trajectories of the favored alleles on each of the 14 chromosomes (with different colors). Column 2 shows the IBD length distribution for two types of IBD segments (red, selection simulations; black, neutral simulations). Column 3 shows the distribution of pairwise genome-wide total IBD sharing. Column 4 shows IBD coverage across the chromosome with each color representing one of 14 chromosomes. Column 5 shows N_e estimates and parameter population size. Error bands incidate 95% confidence intervals. Note that, for axes in Column 2, x axis (IBD length L, bottom) uses a custom scale so that the estimated TMRCA ($50/L$, top) is in a linear scale. For the IBD segment length distribution analysis in column 2, shorter IBD segments (0.2-2 cM) were included in this analysis to cover the more distant past ($>$ 25 generations ago). A length of 0.25, 0.33, 0.5, 1.0 and 100 cM corresponds to a TMRCA of 200, 150, 100, 50 and 0.5 generations ago, respectively. Default selection simulation parameters: selection coefficient $s = 0.3$, selection starting time $g = 80$ generations ago, number of origins of the favored allele $o = 1$. Abbreviations: Neutral, neutral simulation; Selection Orig, positive selection with IBD peak regions not removed; Selection Rmpeaks, positive selection with IBD peak regions removed. Source data are provided as a Source Data file.

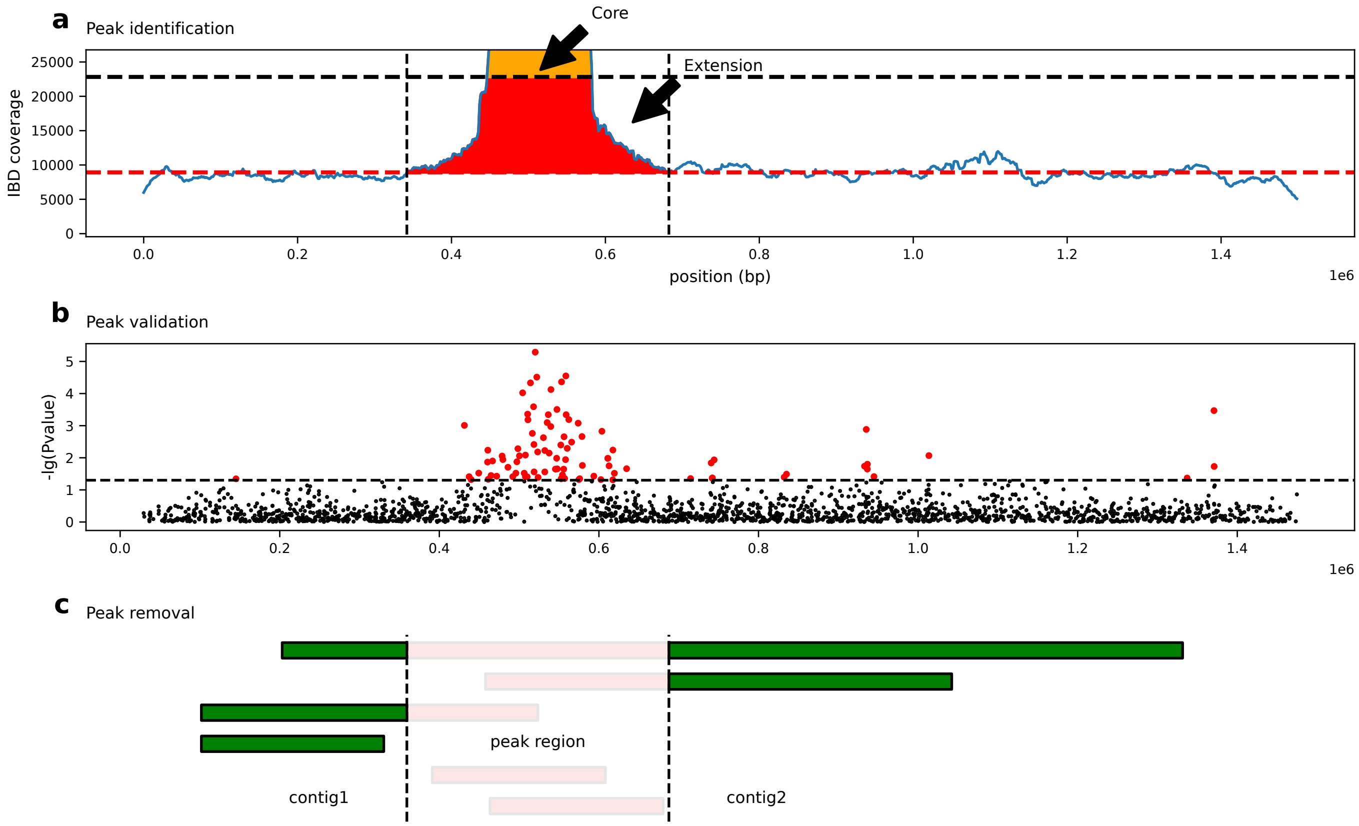

Supplementary Figure 4: Schematic for IBD peak region identification and removal. a, A peak region is a combination of a core region and extension regions. The core region (orange area) is determined by a threshold (black dashed line) of two standard deviations from the chromosomal mean after 5% trimming. This is expanded by extension regions (red area) that extend up to the chromosomal median (red dashed line). b, Peak validation via X_{iHS} scan. Red dots (hits) indicate SNPs with p value < 0.05 based on integrated haplotype score-based X_{iHS} test (See Methods). Peaks that contain at least one hit are kept and treated as validated peaks. c, IBD segments between a pair of isolates (represented by colored bars) are split when they overlap with the peak region (between dotted lines). The segment parts that fall within the peak region (shown in pink) are discarded. This creates a region of with no IBD, and the remaining portion of the chromosome is split into contigs containing IBD segments. These contigs are treated as separate chromosomes for IBDNe inference. Source data are provided as a Source Data file.

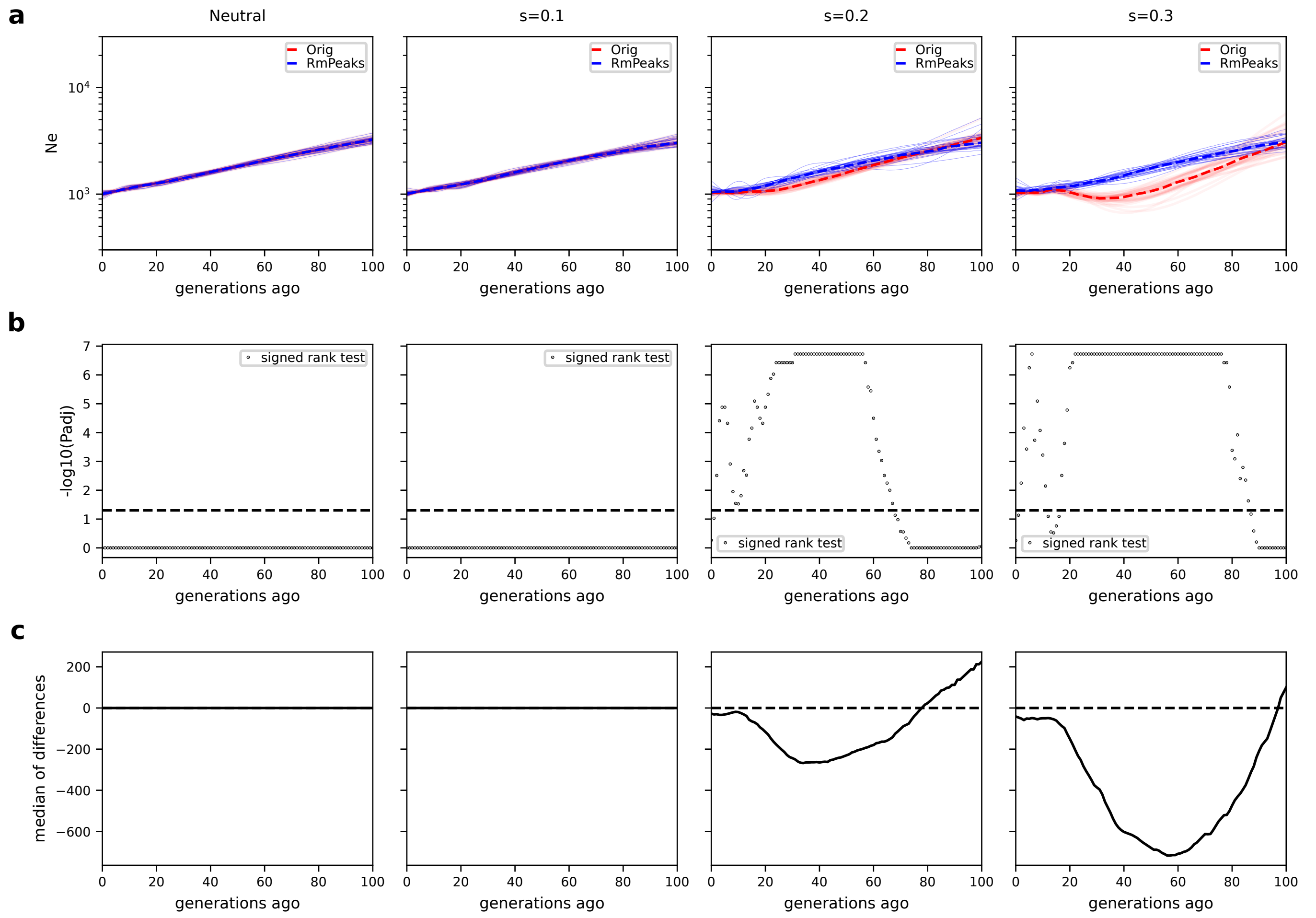

Supplementary Figure 5: Validation of positive selection effects on N_e estimates with replicated simulations. a, Trajectories of estimated N_e. Solid, faint lines are N_e estimates from 30 replicated simulations. Dashed lines are the corresponding medians for each generation. b, Bonferroni-adjusted p values of two-sided signed rank test (exact p values available in the Source Data file) per time point (generation). The tests were run based on log-transformed N_e estimates. The horizontal dashed line represents thresholds for significance ($-\log_{10} 0.05$). c, Differences in N_e estimates (not log-transformed) before and after peak removal. Medians (solid lines) of differences for each time point (over 30 pairs/repeats) were plotted. Dotted lines of $y = 0$ were added for reference. Source data are provided as a Source Data file.

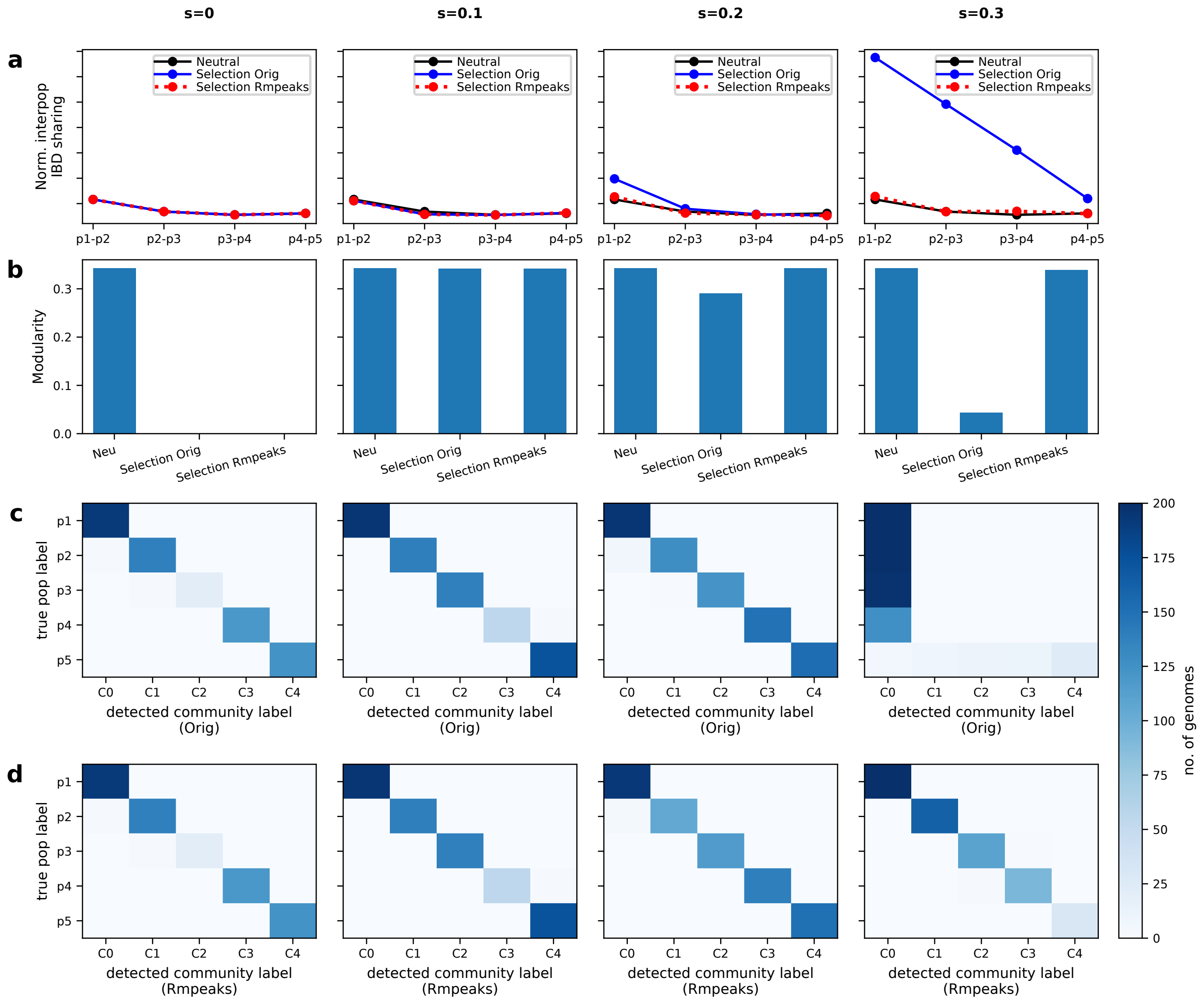

Supplementary Figure 6: The effect of positive selection on IBD-based population structure inference (extending Figure 3). a, Normalized inter-population IBD sharing between nearby demes along the stepping stone chain. From left to right are simulations with different coefficients $s = 0$ (neutral), $s = 0.1$, $s = 0.2$, and $s = 0.3$. b, Modularity of IBD networks with respect to the true population labels under different selection coefficients (neutral, $s = 0.1$, $s = 0.2$, and $s = 0.3$) before and after removing IBD peaks. c-d, IBD network InfoMap community detection before (c) and after (d) removing IBD peaks. For each subplot, rows are true subpopulations labeled as p1-p5 (assigned in simulation), and columns represent the largest 5 detected communities labeled as C0-C4 (with columns reordered to facilitate the comparison of true and inferred labels). The color of each block (see color scale in the color bar) represents the number of genomes/isolates with the given true labels and detected community labels, with darker color indicating more genomes/isolates. Source data are provided as a Source Data file.

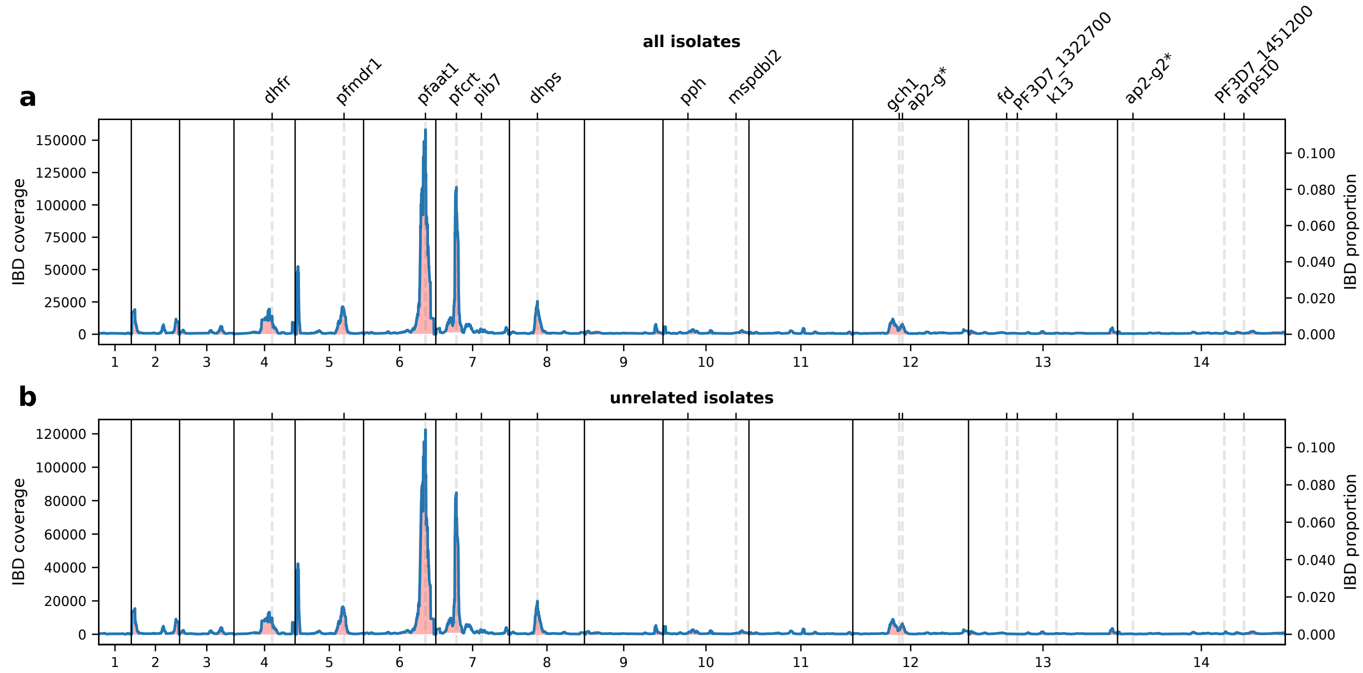

Supplementary Figure 7: IBD coverage profile of all *Pf* isolates and unrelated *Pf* isolates in WAF (in comparison with SEA, Figure 4). a, IBD coverage/proportions of all isolates in WAF (n = 1,674). Labels on the top indicate the center of genes of known or putative drug resistance or that are under selection for sexual commitment (*). b, IBD coverage/proportions of unrelated samples in WAF (n = 1,496). Annotations in (a) are shared with (b); regions with red shading indicate validated peaks (defined in Methods). Note: different scales for y axes (IBD coverage on the left y-axis; IBD proportions on the right y-axis) were used in (a) versus (b) to better reveal the peaks. Source data are provided as a Source Data file.

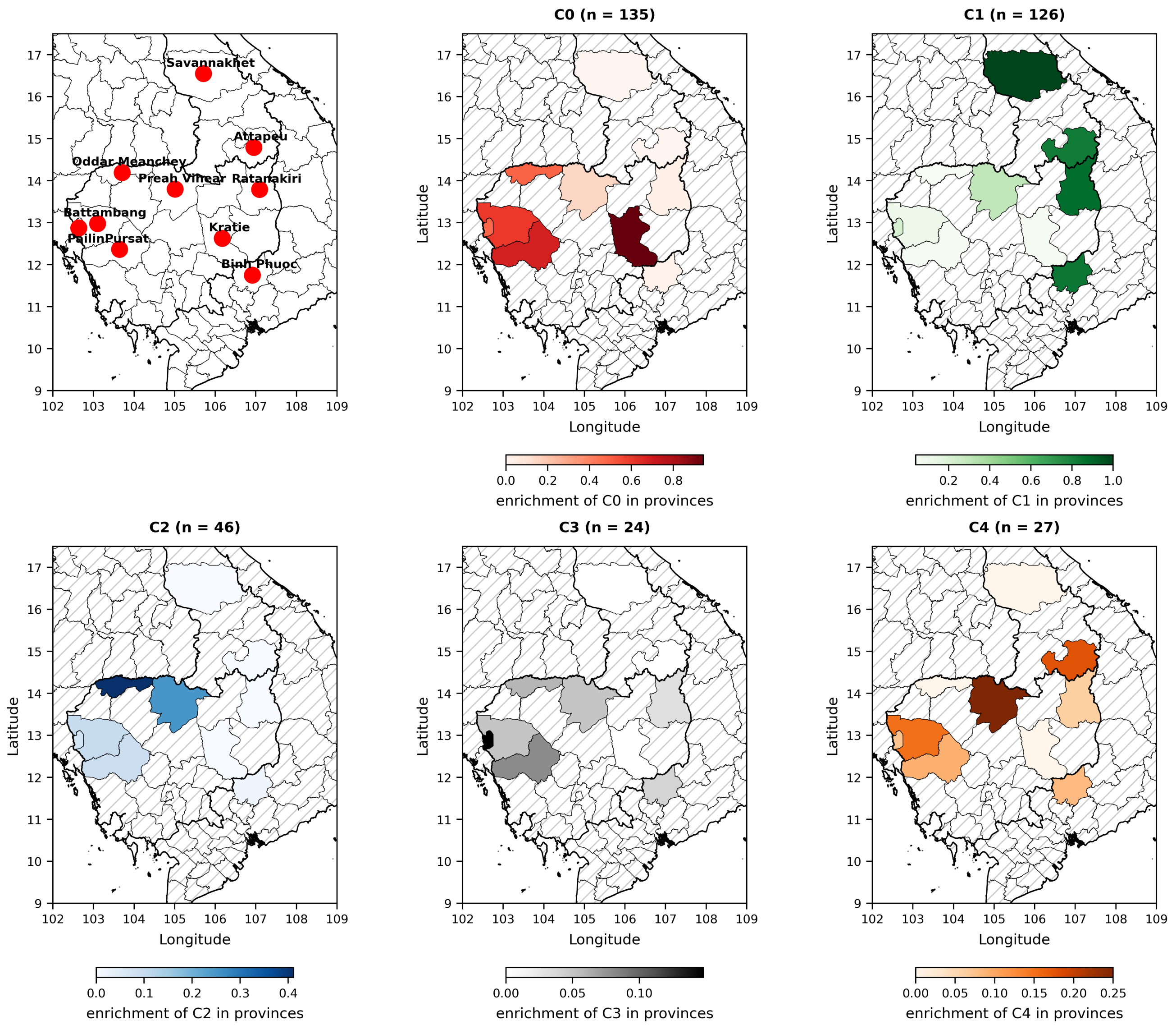

Supplementary Figure 8: Enrichment of the largest 5 SEA *Pf* IBD communities in geopolitically defined regions. The top left panel shows labels for analyzed provinces. The rest of the panels show the enrichment of a given IBD community in different provinces. The enrichment is normalized within each province (the total enrichment of these 5 communities is 100% per province). The darkness of color (see color bars) represents the extent of enrichment of the community in a province. Only provinces with at least 10 samples assigned to these 5 communities are shown. Provinces with fewer than 10 samples assigned to these communities are indicated with hashes. Source Data are provided as a Source Data file. Note: Coordinates of borders around countries or adminstrative regions were obtained from the DIVA-GIS database[4].

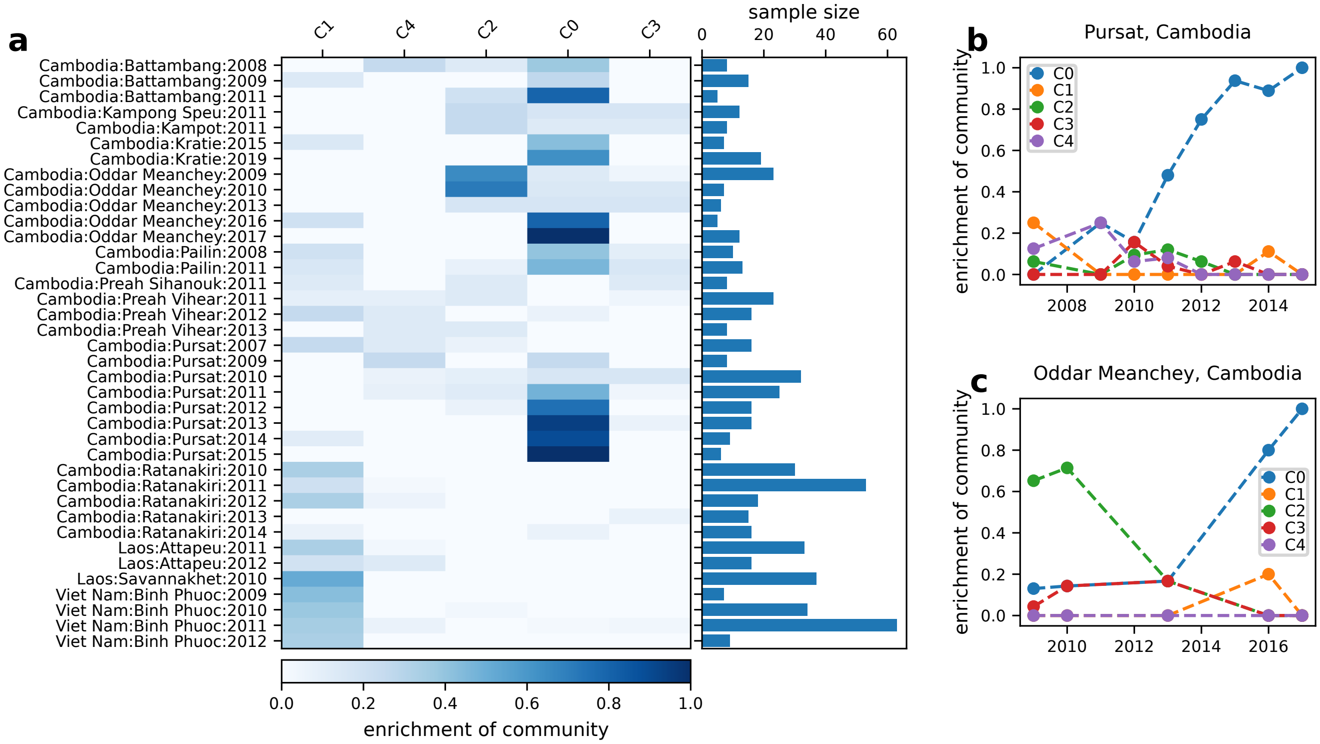

Supplementary Figure 9: Enrichment of the largest 5 SEA communities in each region/year combination. a, Heatmap showing enrichment of top communities in each region-year. The color in the heatmap represents the percentage of samples in a region-year combination (rows) that are assigned to a community (columns). The bar plot on the right of the heatmap shows the number of unrelated samples that are from a region-year (rows). b-c. Dynamics of community enrichment in Pursat (b) and Oddar Meanchey (c) provinces in Cambodia. Source data are provided as a Source Data file.

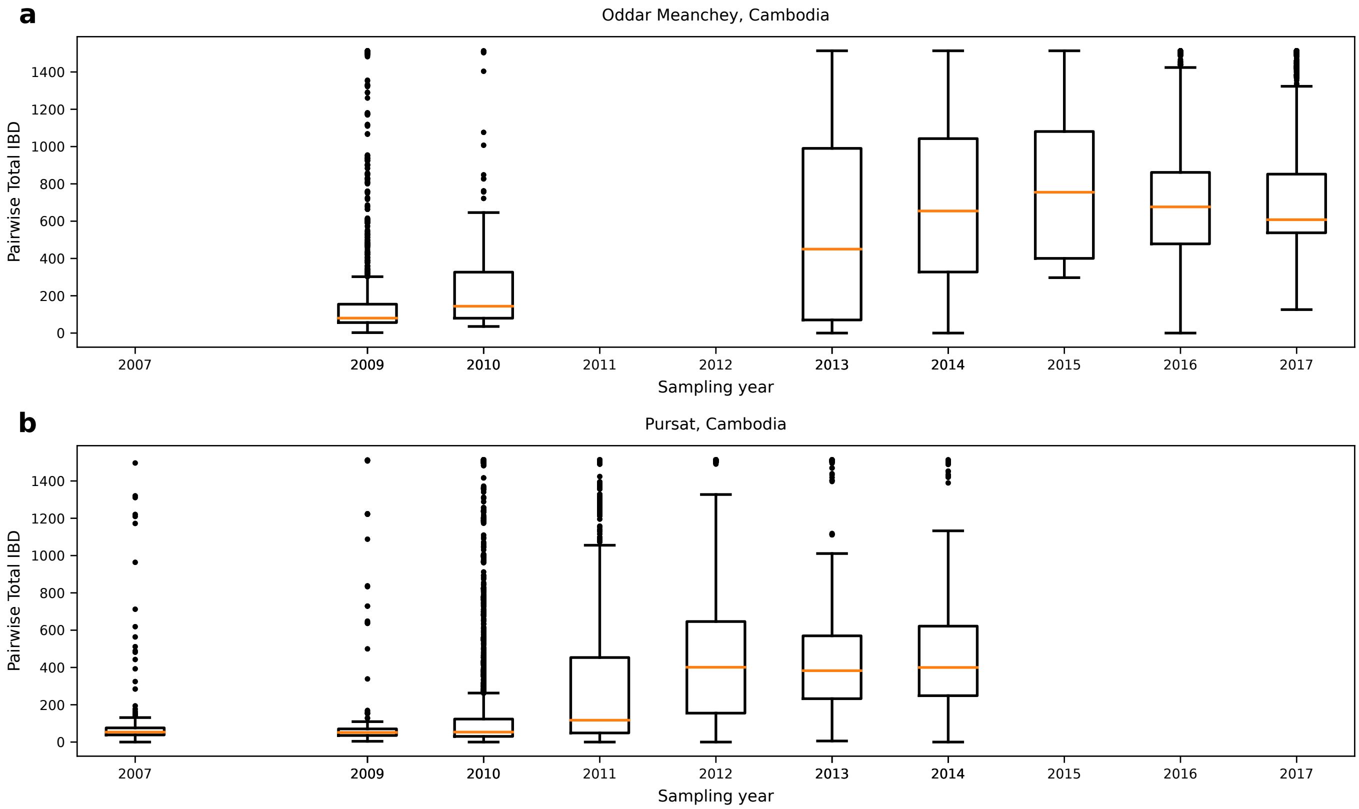

Supplementary Figure 10: Dynamics of pairwise genome-wide total IBD sharing over time. a, Boxplot showing pairwise total IBD in the Oddar Meanchey province of Cambodia. b, Boxplot showing pairwise total IBD in the Pursat province of Cambodia. Note: Each time point represents a year during which at least 30 isolates were collected in the given province (including both related and unrelated isolates). The lower and upper edges of the box are the first and third quartiles of the data per time point. The center lines indicate the medians. The whiskers extend from the box by 1.5x the inter-quartile range. Flier points are those beyond the whisker range. Source data are provided as a Source Data file.

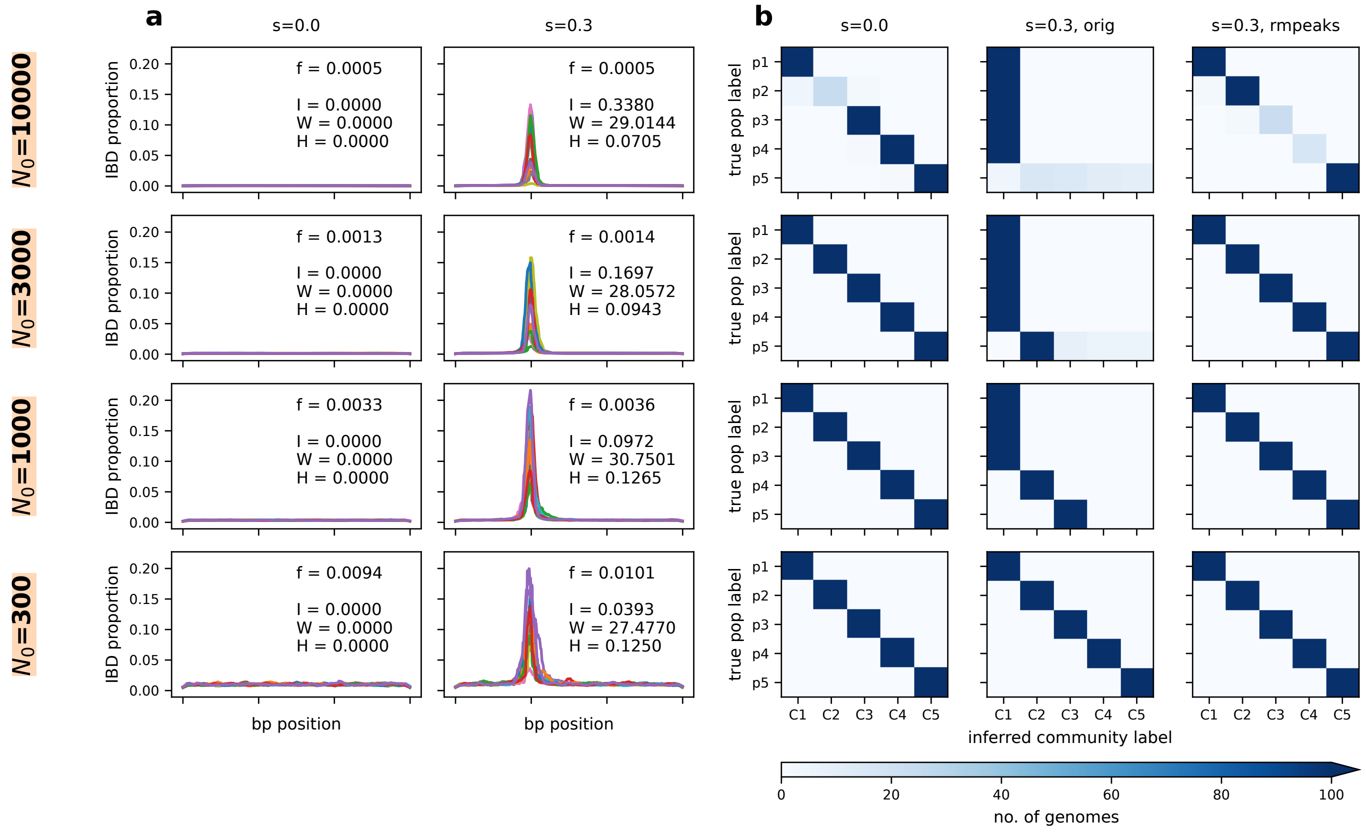

Supplementary Figure 11: Changes in IBD proportions, peak metrics, and population structure inference after modifying the inbreeding potential via tuning present-day population size N_0 in the multiple-population model. a, IBD proportions, estimates of inbreeding potential (f), and peak metrics [average width (W), height (H), and impact index (I)] of multiple-population simulations with different N_0 (rows). Neutral simulations are in the left column and selection simulations are in the right column. For each plot, lines of different colors represent IBD proportions for different chromosomes (14 in total). b, Concordance of true population labels (y-axis) and inferred detected community labels (inferred population labels, x-axis) for neutral (left column), selection (middle column), and selection with peaks removed (right, columns). The color intensity in each block indicates the number of genomes with the given true and inferred labels (also see color bar). For both (a) and (b), N_0 is 10,000, 3,000, 1,000, and 300 for row 1 to row 4 respectively. Abbreviations: Orig, IBD peak regions not removed; Rmpeaks, IBD peak regions removed. Source data are provided as a Source Data file.

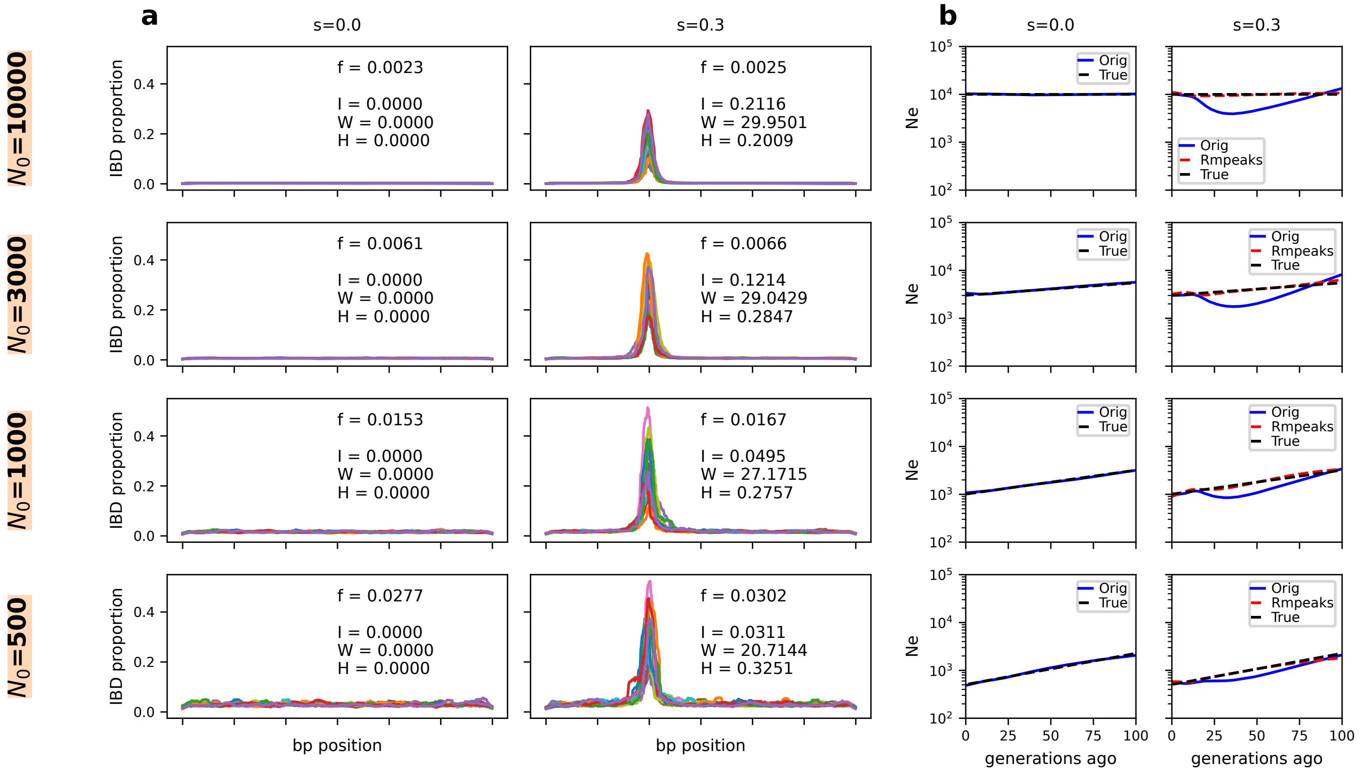

Supplementary Figure 12: Changes in IBD proportions, peak metrics, and N_e estimation after modifying the inbreeding potential via tuning present-day population size N_0 in the single-population model. a, IBD proportions, estimates of inbreeding potential (f), and peak metrics [average width (W), height (H), and impact index (I) across chromosomes] of single-population simulations with different N_0 (rows). Neutral simulations are in the left column and selection simulations are in the right column. For each plot, lines of different colors represent IBD proportions for different chromosomes (14 in total). b, True population size (black dashed line) and inferred N_e before (blue solid line) and after (red solid line) IBD peak removal for neutral (left column) and selection simulations (right column). For both (a) and (b), N_0 is 10,000, 3,000, 1,000, and 500 for row 1 to row 4 respectively. Error bands indicate 95% confidence intervals. Abbreviations: Orig, with IBD peak regions not removed; Rmpeaks, with IBD peak regions removed. Source data are provided as a Source Data file.

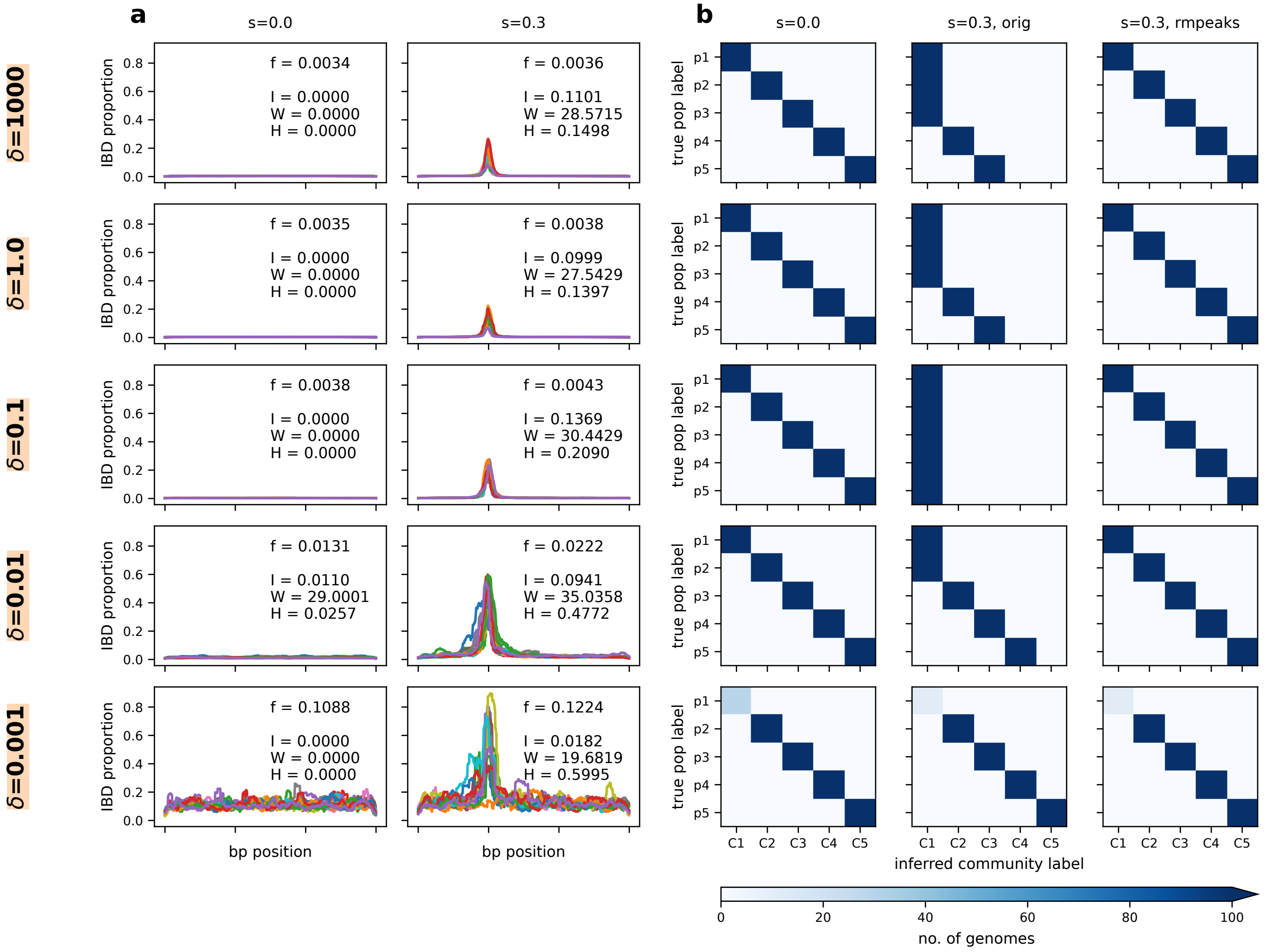

Supplementary Figure 13: Changes in IBD proportions, peak metrics, and population structure inference after modifying the inbreeding potential via tuning assortative mating parameter δ (defined in Supplementary Note 2) in the multiple-population model. a, IBD proportions, estimates of inbreeding potential (f), and peak metrics [average width (W), height (H), and impact index (I)] of multiple-population simulations with different δ (rows). Neutral simulations are in the left column and selection simulations are in the right column. For each plot, lines of different colors represent IBD proportions for different chromosomes (14 in total). b, Concordance of true population labels (y-axis) and inferred detected community labels (inferred population labels, x-axis) for neutral (left column), selection (middle column), and selection with peaks removed (right, columns). The color intensity in each block indicates the number of genomes with the given true and inferred labels (also see color bar). For both (a) and (b), δ is 1,000 (control), 1.0, 0.1, 0.01, and 0.001 for rows 1 to 5 respectively. Abbreviations: Orig, IBD peak regions not removed; Rmpeaks, IBD peak regions removed. Source data are provided as a Source Data file.

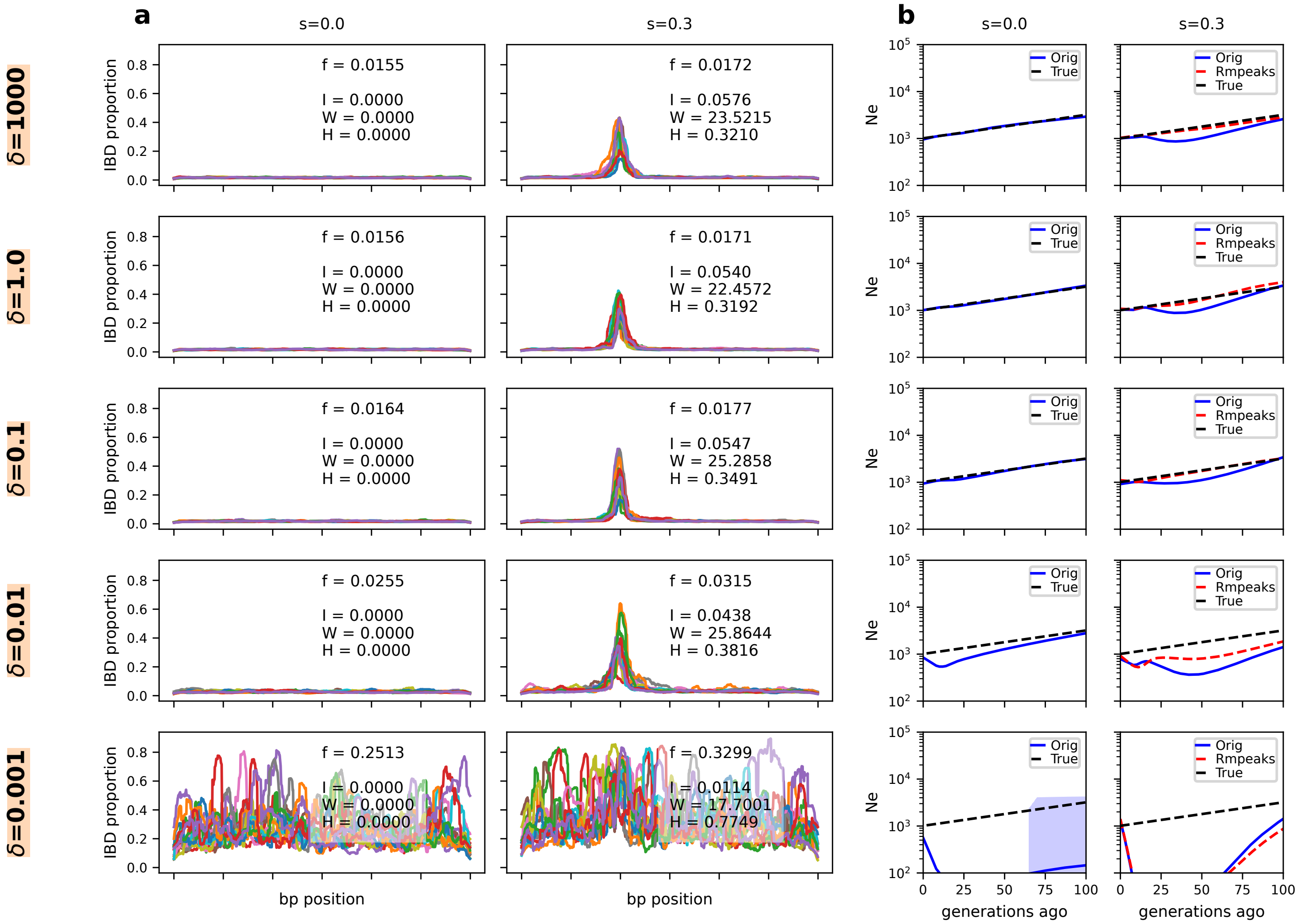

Supplementary Figure 14: Changes in IBD proportions, peak metrics, and N_e estimation after modifying the inbreeding potential via setting different values for the assortative mating parameter δ in the single-population model. a, IBD proportions, estimates of inbreeding potential (f), and peak metrics [average width (W), height (H), and impact index (I) across chromosomes] of single-population simulations with different δ (rows). Neutral simulations are in the left column, and selection simulations are in the right column. For each plot, lines of different colors represent IBD proportions for different chromosomes (14 in total). b, True population size (black dashed line) and inferred N_e before (blue solid line) and after (red solid line) IBD peak removal for neutral (left column) and selection (right column) simulations. Error bands indicate 95% confidence intervals. For both (a) and (b), δ is 1,000 (control), 1.0, 0.1, 0.01, and 0.001 for rows 1 to 5 respectively. Abbreviations: Orig, IBD peak regions not removed; Rmpeaks, IBD peak regions removed. Source data are provided as a Source Data file.

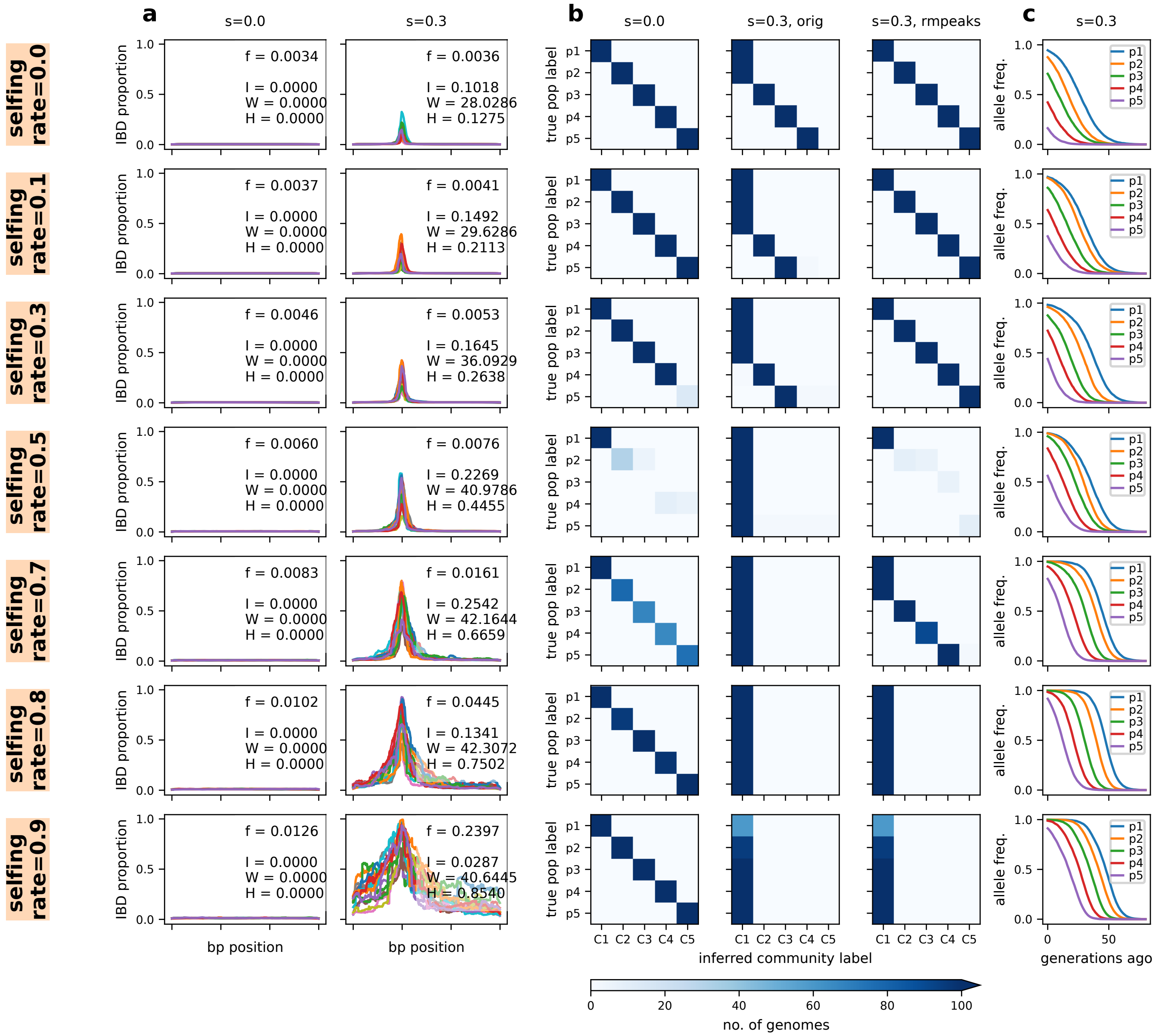

Supplementary Figure 15: Changes in IBD proportions, peak metrics, and population structure inference after modifying the inbreeding potential via tuning the selfing rate in the multiple-population model. a, IBD proportions, estimates of inbreeding potential (f), and peak metrics [average width (W), height (H), and impact index (I)] of multiple-population simulations with different selfing rates (rows). Neutral simulations are in the left column and selection simulations are in the right column. For each plot, lines of different colors represent IBD proportions for different chromosomes (14 in total). b, Concordance of true population labels (y-axis) and inferred detected community labels (inferred population labels) for neutral (left column), selection (middle column), and selection with peaks removed (right, columns). The color intensity in each block indicates the number of genomes with the given true and inferred labels (also see color bar). c, Allele frequency of the allele under positive selection. For each subpopulation (p1 to p5), the curve represents the average allele frequency of selected alleles across chromosomes. For (a-c), the selfing rate is 0.0 (control), 0.1, 0.3, 0.5, 0.7, 0.8, and 0.9 for rows 1 to 7 respectively. Abbreviations: Orig, with IBD peak regions not removed; Rmpeaks, with IBD peak regions removed. Source data are provided as a Source Data file.

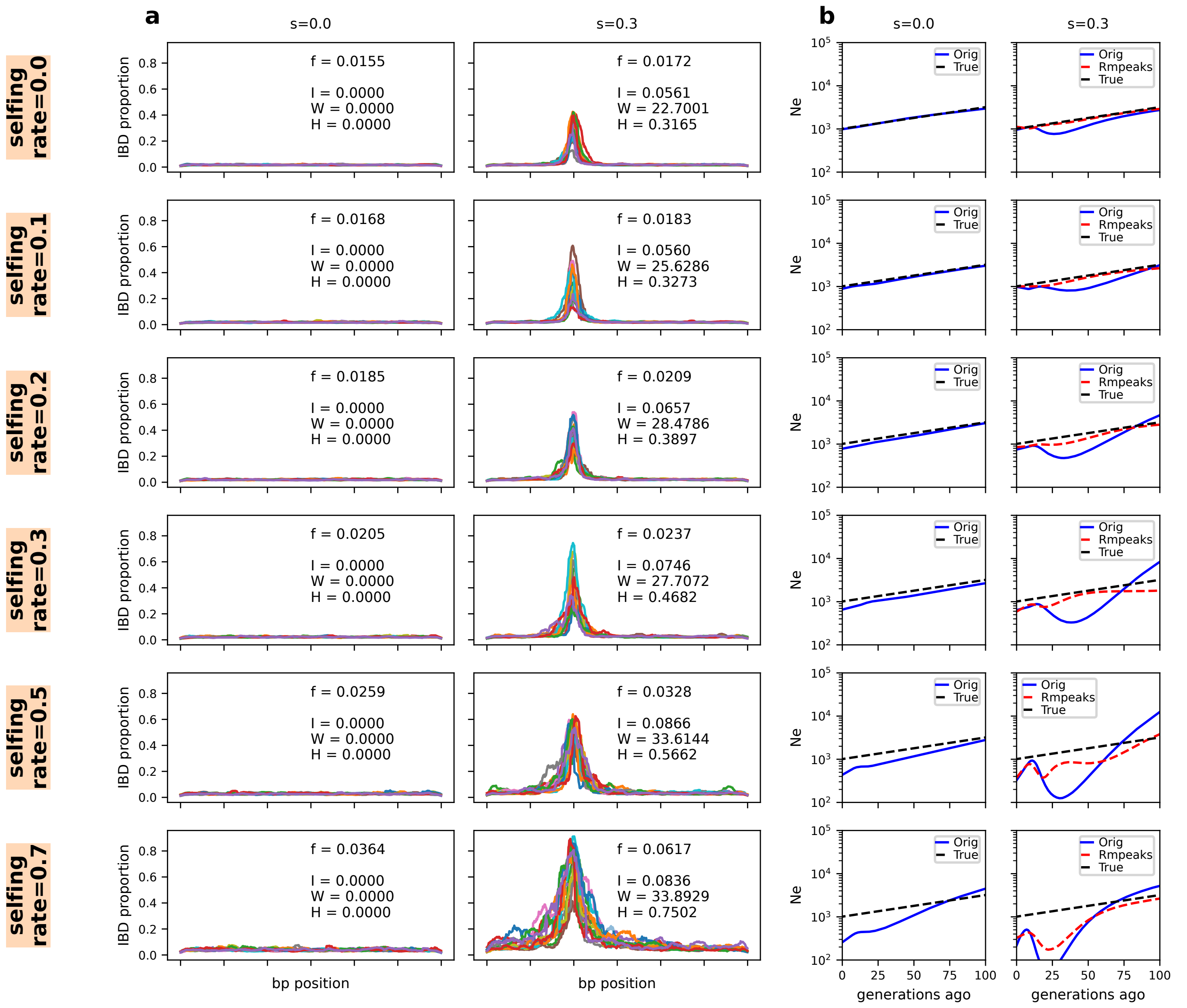

Supplementary Figure 16: Changes in IBD proportions, peak metrics, and N_e estimation after modifying the inbreeding potential via tuning the selfing rate in the single-population model. a, IBD proportions, estimates of inbreeding potential (f), and peak metrics [average width (W), height (H), and impact index (I) across chromosomes] of single-population simulations with different selfing rates (rows). Neutral simulations are in the left column, and selection simulations are in the right column. For each plot, lines of each different color represent IBD proportions for different chromosomes (14 in total). b, True population size (black dashed line) and inferred N_e before (blue solid line) and after (red solid line) IBD peak removal for neutral (left column) and selection (right column) simulations. Error bands indicate 95% confidence intervals. For both (a) and (b), the selfing rate is 0.0 (control), 0.1, 0.3, 0.5, 0.7 for rows 1 to 6 respectively. Abbreviations: Orig, with IBD peak regions not removed; Rmpeaks, with IBD peak regions removed. Source data are provided as a Source Data file.

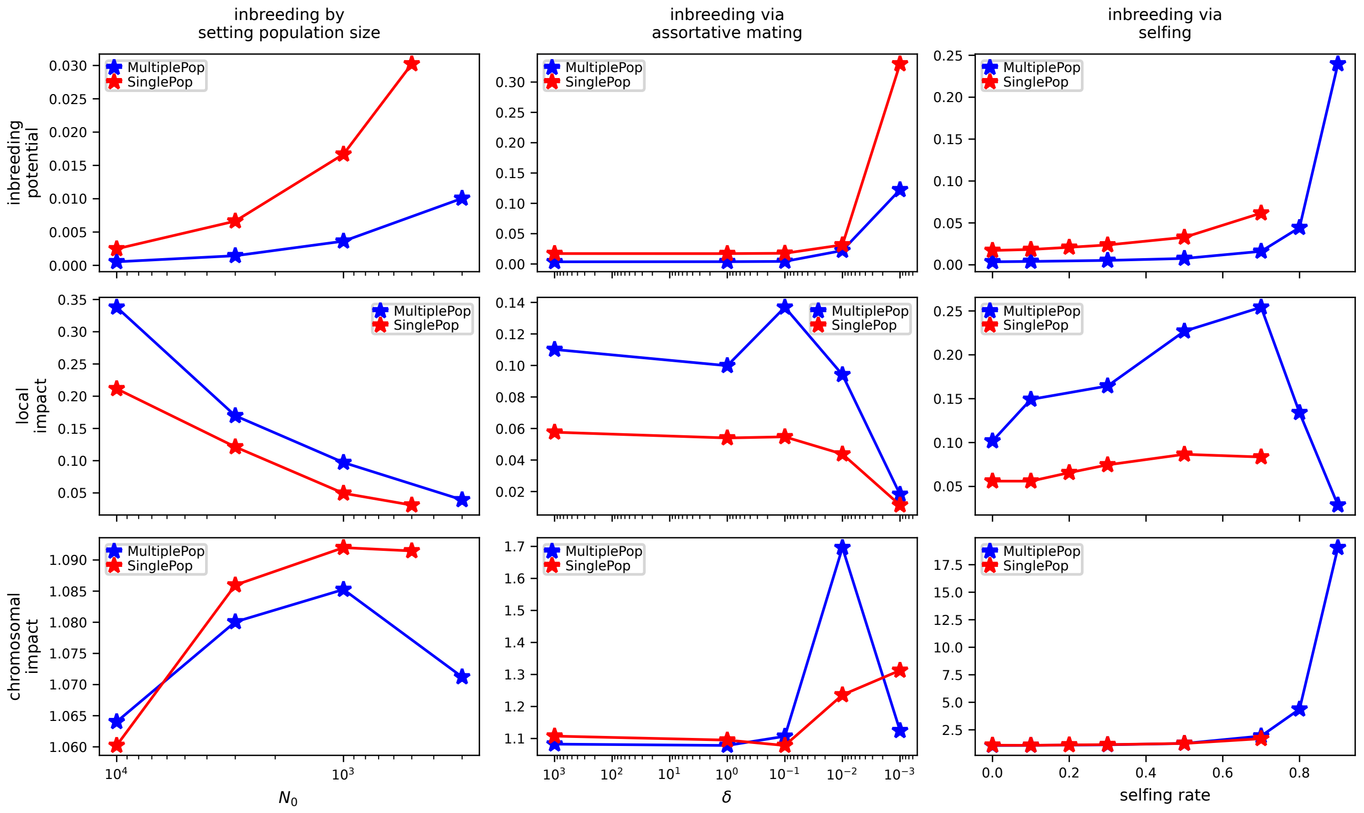

Supplementary Figure 17: Overlay of inbreeding potential estimates, local impact indices, and global impact indices across various inbreeding simulations. Inbreeding simulations mediated by shrinking population size (Supplementary Figure 11-12, summarized in column 1 here), assortative mating (Supplementary Figure 13-14, summarized in column 2 here), and selfing (Supplementary Figure 15-16 summarized in column 3 here) are summarized for both single (red) and multiple (blue) population models. Estimates of inbreeding potential are plotted in the top row, local impact (mean peak impact index) in the middle row, and global/chromosomal impact (global impact index) in the bottom row. Source data are provided as a Source Data file.

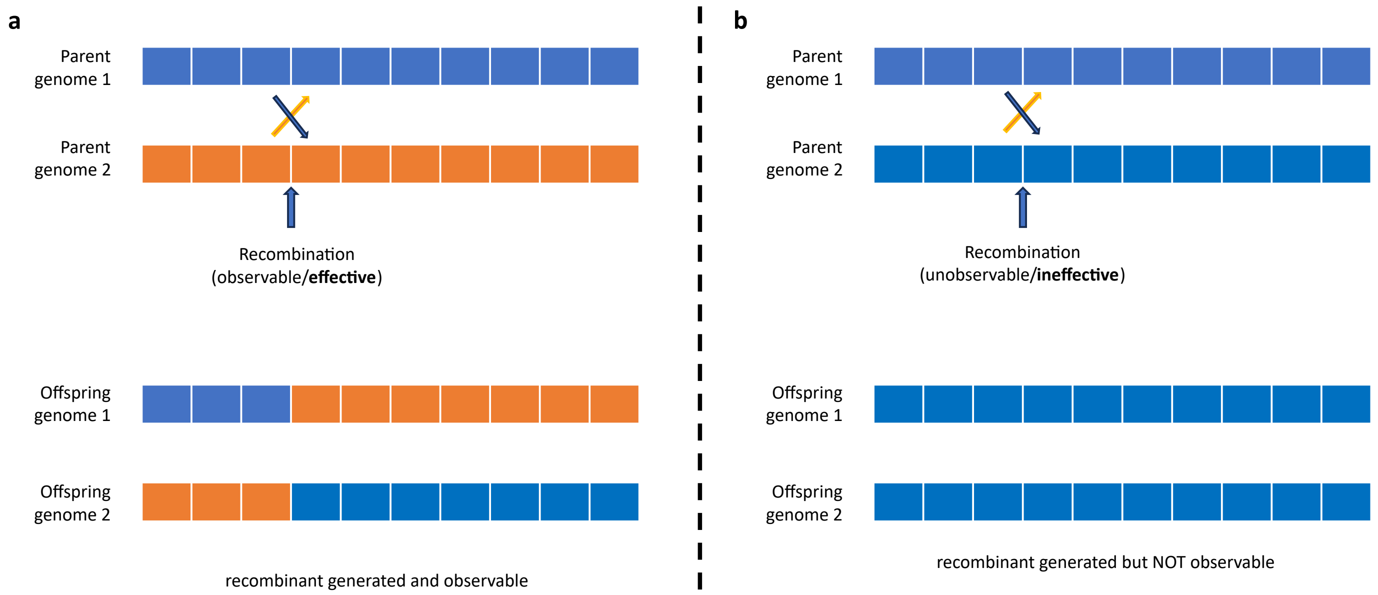

Supplementary Figure 18: The true recombination rate and effective recombination rate. a, Recombination between genetically distinct genomes. b, Recombination between genetically identical genomes. See Supplementary Note 2 Section 2 for detailed description.

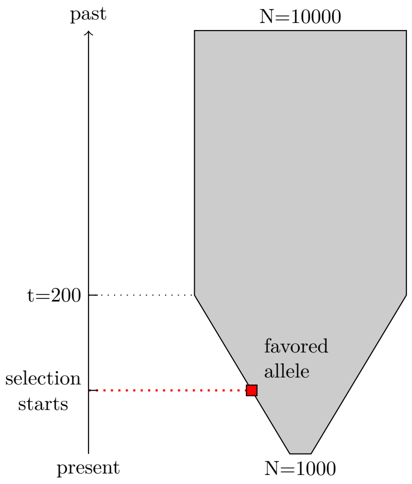

Supplementary Figure 19: Schematic for demographic model with shrinking population size in recent history. See Supplementary Note 2 Section 4 for detailed description.

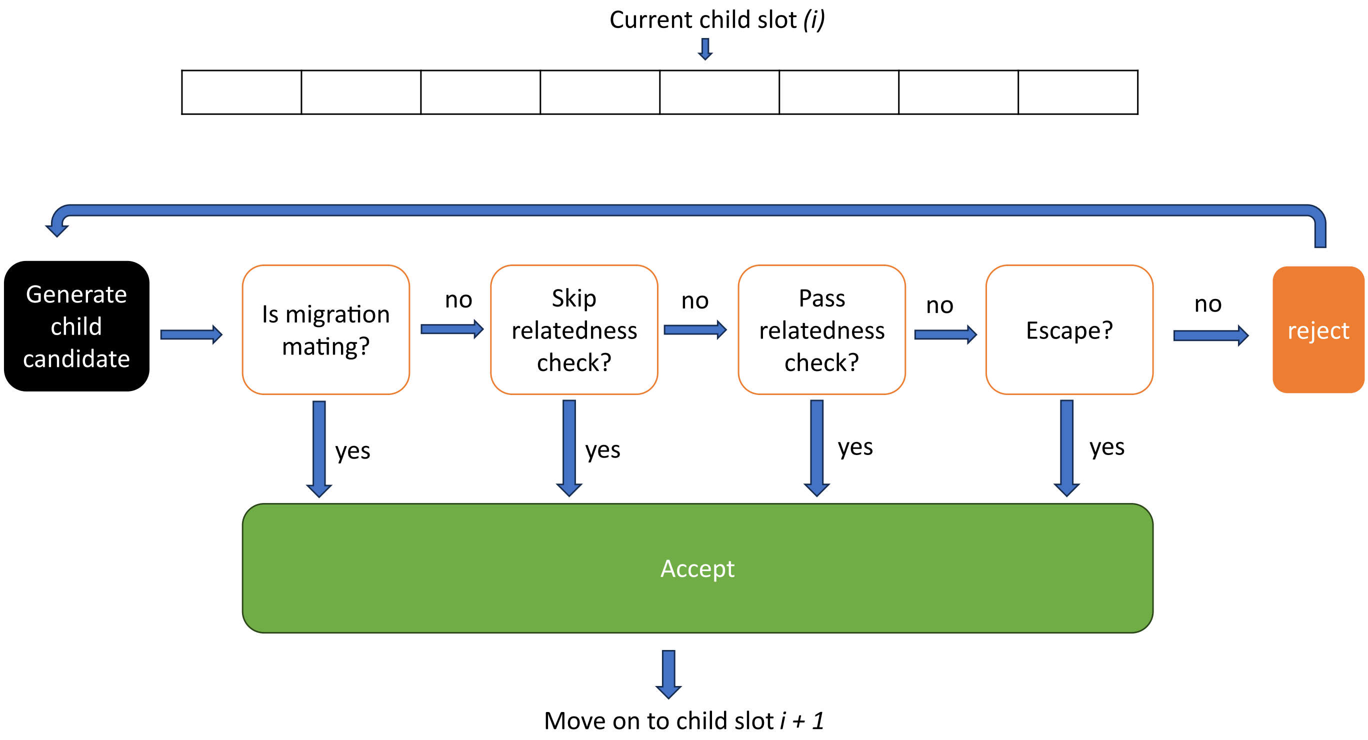

Supplementary Figure 20: modifyChild logic for accepting and rejecting child candidates for each child slot. See Supplementary Note 2 Section 4 for detailed description.

Supplementary Tables

Supplementary Table 1: The effects of positive selection on IBD-based population assignments and the benefits of selection correction are selection-strength dependent (based on simulation). For each parameter (neutral, $s = 0.1$, $s = 0.2$, and $s = 0.3$), $n = 30$ independent simulations were performed. Adjusted Rand Scores were presented as mean ± standard deviation. p-values were determined by two-sided paired t-tests. Abbreviations: Orig, with IBD peak regions not removed; Rmpeaks, with IBD peak regions removed. Source data are provided as a Source Data file.

Group	Adjusted Rand (Orig)	Adjusted Rand (Rmpeaks)	p-value(t-test)
Neutral	0.614 ± 0.066	0.614 ± 0.065	1.000
$s = 0.1$	0.631 ± 0.056	0.631 ± 0.056	1.000
$s = 0.2$	0.507 ± 0.063	0.629 ± 0.065	0.000
$s = 0.3$	0.107 ± 0.018	0.582 ± 0.084	0.000

Supplementary Table 2: Estimating uncertainty in population structure inference for empirical data based on Jackknife resampling over chromosomes. Columns 1-8 show details of community detection results in each Jackknife sampling. The last column shows whether selection correction significantly changes IBD-based community detection in two transmission settings. Orig/Rmpeaks, results for IBD segments before and after removing peaks. NComm, total number of communities inferred. TopComm, the size (number of samples) in the largest 5 communities. AdjRand, adjust rand score based on samples that exist in the top 5 communities of both Orig and Rmpeaks results. RmChr, the chromosome number was removed in Jackknife resampling. Q3, the third quartile; IQR, interquartile range, Q3 - Q1. Note: Selection correction resulted in statistically significant changes in population structure inference for both SEA and WAF regions; however, SEA's adjusted rand indices between population labels before versus after correction was very close to 1, suggesting the correction effect in SEA was very small; that for WAF was much smaller than 1, indicating large correction effect in WAF.

		**Orig**		**Rmpeaks**				
Region	N	NComm	TopComm	NComm	TopComm	AdjRnd	RmChr	Summary
SEA	701	75	135,126,46,27,24	78	136,113,45,25,23	0.977	None	
SEA	701	76	134,132,44,24,24	80	136,103,43,25,23	0.985	1	
SEA	701	75	139,131,43,25,25	79	136,116,43,25,24	0.990	2	
SEA	701	74	139,126,45,27,24	77	137,120,42,27,24	0.961	3	
SEA	701	74	140,132,42,25,24	77	137,120,43,25,24	0.980	4	Median: 0.977
SEA	701	73	141,139,44,26,23	79	136,117,45,25,21	0.973	5	
SEA	701	73	137,129,46,24,24	75	136,119,42,27,22	0.973	6	
SEA	701	74	139,128,43,24,23	76	139,110,43,23,20	0.979	7	$Q_3 + 1.5 \times$ IQR: 0.992
SEA	701	74	139,135,44,27,23	77	140,119,42,28,26	0.990	8	
SEA	701	78	137,127,43,24,23	78	137,116,41,24,24	0.974	9	
SEA	701	77	136,136,42,24,23	82	136,126,42,27,21	0.952	10	Significant: true
SEA	701	75	134,115,45,24,24	77	133,124,43,25,23	0.979	11	
SEA	701	72	134,127,44,28,23	75	137,105,41,29,28	0.972	12	
SEA	701	75	134,119,45,27,26	75	135,112,44,29,24	0.974	13	
SEA	701	76	143,117,40,24,24	78	138,111,32,26,22	0.981	14	
WAF	1496	75	1222,25,17,16,11	189	59,36,36,35,33	0.093	None	
WAF	1496	74	1221,25,17,16,12	187	64,37,33,32,31	0.090	1	
WAF	1496	74	1225,24,17,16,12	187	59,38,35,32,31	0.088	2	
WAF	1496	71	1228,24,17,16,14	188	60,39,39,39,33	0.070	3	
WAF	1496	76	1223,25,17,16,12	187	58,50,35,34,33	0.090	4	Median: 0.088
WAF	1496	73	1223,25,18,18,11	181	80,62,53,36,33	0.076	5	
WAF	1496	83	1170,27,25,19,11	183	61,43,37,33,32	0.085	6	
WAF	1496	75	1219,25,18,16,12	199	46,35,32,30,27	0.158	7	$Q_3 + 1.5 \times$ IQR: 0.117
WAF	1496	71	1229,25,17,16,11	183	66,40,39,38,36	0.083	8	
WAF	1496	74	1230,24,16,13,12	184	69,36,36,33,27	0.091	9	
WAF	1496	69	1238,24,16,14,12	188	59,57,43,39,31	0.068	10	Significant: true
WAF	1496	74	1230,24,17,16,12	194	56,40,35,34,28	0.068	11	
WAF	1496	67	1238,25,16,16,12	194	61,40,36,35,31	0.135	12	
WAF	1496	69	1237,25,17,15,14	191	66,39,38,36,36	0.088	13	
WAF	1496	71	1233,25,19,14,12	184	62,38,36,34,34	0.000	14	

Supplementary Notes

Plasmodium falciparum parasites, especially those from low transmission settings, often undergo pervasive inbreeding, because of the fusion/mating between gametes with high genetic relatedness. The high level of inbreeding is accompanied by a low effective recombination rate, a slow decay of linkage disequilibrium, and a profound hitchhiking effect. Thus, the level of inbreeding can dramatically affect the magnitude of selection-induced bias on IBD-based inferences. However, a quantitative analysis of the relationship between inbreeding and selection bias is lacking. Here, we provided (1) definition of quantitative metrics to assess the amount of inbreeding and the extent of selection impact, (2) detailed methods for simulating different types and levels of inbreeding, and (3) results on how different levels of inbreeding affect the extent of selection bias and the necessity for peak-removal based selection correction.

Supplementary Note 1 Metrics Used to Analyze Inbreeding and Selection Effects

1 Inbreeding potential

To estimate inbreeding levels in haploid genomes, we use a post-hoc measure termed inbreeding potential. This is calculated from each simulation by first obtaining true Identity-by-Descent (IBD) information using the tool tskibd. We then determine IBD coverage and IBD sharing proportions at evenly spaced sampling points along a chromosome, with each point spaced 0.1 cM apart. The inbreeding potential is estimated by averaging the IBD sharing proportions across all these points, except those within IBD peak regions. This approach ensures that our measure reflects baseline inbreeding levels, unaffected by local changes due to selection. Mathematically, the inbreeding potential is approximated as follows:

$$f = \frac{\sum_{i \in S_{\mathrm{non-peak}}} f_i}{|S_{\mathrm{non-peak}}|}$$

Here, f_i is the IBD sharing proportion at a given sampling point i, that is, the total number of IBD segments shared by any pair of haploid genomes in the population that contain this sampling point (IBD coverage over the sampling point) divided by the number of all possible pairs of haploid genomes in the population; $S_{\mathrm{non-peak}}$ denotes the set of sampling points that are not located within any peak regions; $|S|$ means the number of elements in the set S. We prefer the term inbreeding potential over inbreeding coefficient because the latter typically applies to diploid individuals, where estimating the inbreeding coefficient is straightforward based on IBD within the individual[5]. However, for haploid genomes that we obtained from human blood samples representing monoclonal infections or polyclonal infections with a predominant clone, information on which haploids combine into a transient diploid in the mosquito is unavailable, making the diploid concept of the inbreeding coefficient less applicable. Therefore, using the average IBD sharing proportion as a measure of inbreeding potential is both practical and relevant for exploring the effects of selection on IBD patterns and the inference of effective population size and population structure. This measure, termed as inbreeding potential, essentially represents the average inbreeding coefficients in offspring when parents of each child are randomly drawn from the parent generation.

2 Peak impact index

The peak impact index is a metric we proposed to further distinguish noise from true peaks due to strong selection and quantify the necessity for peak removal-based selection correction. It is calculated as the ratio of the increase in IBD attributable to a specific peak to the total IBD that would be observed if the peak regions were instead neutral. The formula for the peak impact index is as follows:

$$I_k = \frac{\sum_{i \in S_{\mathrm{peak}_k}} (f_i - f)}{f * |S_{\mathrm{all}}|}$$

Here, S_{peak_k} is the set of sampling points located within the peak region k, and S_{all} is the set of all sampling points. We found via a series of simulations that the peak impact index is $\ll 0.01$ for noisy peaks – the

small/false positive peaks in both neutral and weak($s = 0.15$)/strong($s = 0.3$) selection simulations, whereas the peak impact indices are > 0.01 for true peaks (peaks representing sites under selection). Therefore, a threshold of 0.01 for the peak impact index serves as a criterion for identifying peaks that warrant selection correction by peak removal. Moreover, the peak impact index provides a quantitative means to assess the degree of selection bias at the IBD level. A higher peak impact index indicates a more significant (local) selection bias due to a particular peak, suggesting a greater need for correction. Further details on this, especially concerning our inbreeding simulations, are discussed below (see Supplementary Note 3).

3 Width of peak region

The metric width of peak region is designed to approximate the extent of genomic regions locally affected by selection. For example, a selective sweep with a broader (non-global) impact is expected to result in a larger value for this metric. This width is calculated as the difference between the starting and ending coordinates of a peak region, identified using our peak-identification algorithm as detailed in Methods. This approach provides a straightforward and quantifiable measure of the spatial extent of selection's local influence on a chromosome. Note: as the current method of determining the peak coordinates is heuristic and the IBD coverage/proportion curve is not smoothed, there is a potential for underestimation of peak width when the IBD coverage curve has large variation due to high inbreeding.

4 Global (Chromosomal) impact index

The global (chromosomal) impact index is a metric we developed to assess the chromosome-wide effect of positive selection on a chromosome as opposed to the local impact. It is calculated as the ratio of the inbreeding potential estimated from a positive selection simulation to that of a corresponding neutral simulation:

$$I^G = \frac{f_{\text{selection}}}{f_{\text{neutral}}}$$

Here, $f_{\text{selection}}$ is the baseline inbreeding potential (as defined above) for a selection simulation, and f_{neutral} is that of a corresponding neutral simulation, which shares all simulation parameters except the selection parameters. This index effectively captures the chromosome-wide impact of a strong selective sweep; a larger value indicates a more pronounced global effect of selection. It's important to note that the global impact index is specifically designed for simulation analyses, as it requires neutral control for comparison.

Supplementary Note 2 Inbreeding Modeling Strategies

1 Simplified inbreeding simulation

Modeling inbreeding can be extremely difficult given the complex life cycle of *P. falciparum*. Realistic inbreeding modeling would require multiple levels of control: inbreeding within mosquitos, inbreeding due to the division of the oocyst population into individual mosquitoes, and, inbreeding due to geographic isolation[6]. Inbreeding simulation with this level of complexity is beyond the scope of our current study. Here, we modeled inbreeding with simplified assumptions: we assumed a Wright-Fisher population of hermaphrodite diploids, with two haploid genomes corresponding to each present-day diploid. The simplified model may be somewhat artificial; however, it can still be utilized to explore how different levels of inbreeding influence the effect of strong positive selection on IBD-based inferences of demography and population structure.

2 True versus effective recombination rates

As mentioned above, high inbreeding is associated with low effective recombination rate. There is a significant distinction between the parameter/true recombination rate and the effective recombination rate. The true recombination rate (specified as a recombination parameter in simulations) measures the rate of actual recombination events, whether an observable recombinant is formed or not. We explain this by looking at two extreme cases (Supplementary Figure 18): In case A, recombination occurs between two distinct haploid

genomes (top two genomes in panel a) with completely different DNA sequences (as indicated by blue versus brown colors). The recombination is observable as the offspring genomes are recombination/mixture of parts of the parent genomes. We call this recombination event effective and observable considering that we can infer the recombination event by comparing offspring genomes with parent genomes. However, in case B, recombination happens between two identical haploid genomes (top two genomes in panel b) with identical DNA sequences. The recombination is not observable as the offspring genomes are identical to each other and the parents' genomes. We call this recombination event ineffective and unobservable. In our simulations, the recombination rate parameter (6.67×10^{-7} per generation per bp, or 15 kb/cM) refers to the true recombination rate, which accounts for all recombination events, including those unobservable due to genetic identity between the recombining genomes. The effective recombination rate is not explicitly specified in the simulation but can be controlled by setting the true recombination rate and adjusting parameters to fine-tune the level of inbreeding.

3 Inbreeding coefficient cannot be directly specified but can be indirectly modified

Although SLiM[7,8] does not offer direct control over the inbreeding coefficient, in our analyses, we effectively modeled inbreeding through parameters influencing population size, assortative mating, and selfing, as noted above and described in more detail below. Our strategy involved adjusting these parameters based on post-simulation inbreeding potential estimates (defined in Supplementary Note 1) to assess different inbreeding levels.

4 Three strategies to modify inbreeding levels

We modeled inbreeding via three different strategies in both the single- and multiple-population models, to provide a more comprehensive understanding of how different levels and types of inbreeding affect the magnitude of selection bias:

Modifying the inbreeding potential by reducing population size We set population size as a function of the generation time and called the setSubpopulationSize function in the SLiM script for each generation (mating is random within each subpopulation). For example, to simulate a shrinking population size from a size 200 generations ago $N_{200} = 10{,}000$ to a size at present $N_0 = 1{,}000$, we set population size

$$N_t = N_0 \left(\frac{N_{200}}{N_0} \right)^{t/200}$$

for any generation between 0-200 generations ago (Supplementary Figure 19). We experimented with different N_0 values such as 10,000, 3,000, 1,000, and 500, enabling us to explore a range of inbreeding potentials (a proxy metric to measure inbreeding level for haploid genomes, see definition above). For the single-population model, the minimum N_0 is 500 diploids as we aim to sample 1000 haploid genomes. For the multiple-population model, we do the same to each subpopulation but with N_0 values no smaller than 200 haploid genomes, as we aim to sample a total of 1,000 from the five subpopulations. In this type of inbreeding, we only changed the value of the parameter N_0 (as N_t for $t = 0$ to 200 can be calculated from N_0 and N_{200}). We kept values for the rest of the parameters the same with the detailed values of all parameters for each simulation available from our GitHub repository (`https://github.com/bguo068/posseleff_simulations/blob/main/simulations/Readme.md`).

Modifying the inbreeding potential by simulating different levels of positive assortative mating We employ the modifyChild callback for modeling positive assortative mating, preferring it over the mateChoice callback due to its flexibility and computational efficiency in our scenarios. Our implementation of the modifyChild callback in SLiM involves several steps (Supplementary Figure 20):

1. Child candidate generation: For child slot i, SLiM internally generates a child candidate, provides information about both the child and parents, and enters the modifyChild callback if the backward generation time < parameter G.

2. Migration mating: For the multiple-population model, if the parents of the child candidates are migrants from other subpopulations, the relatedness between the two parents is mostly likely to be very low, causing a high rejection rate and ineffective migration. We use this check step so that any migration mating is accepted regardless of relatedness. Note that the shortcut only happens in the first entry of the callback for each child slot so that inbreeding modeling integrates better with migration.

3. Bypass logic: we provide a bypass logic to avoid reaching extreme inbreeding in a short time window otherwise most of the simulated genomes would be removed due to high genetic relatedness (defined as pairwise IBD > 0.5 genome size), making IBD-based N_e inferences infeasible. For this step, we provide two parameters, bypass factor B (integers > 1) and modifier C. When $C = 0$, only a fraction of $1/B$ enters the next step (relatedness checking step), and a chance of $1 - 1/B$ that the child candidate will be directly accepted. When $C = 1$, a factor of $1 - 1/B$ enters the next step, and a probability of $1/B$ is directly accepted.

4. Relatedness checking: if the parents share a high genetic relatedness, the child candidate will be immediately accepted. We provide parameter D to set the relatedness threshold. Any offspring with parent relatedness $\geq 1/D$ will be accepted, otherwise, enter the next step.

5. Escape logic: The previous step promotes inbreeding by accepting offspring with high parent relatedness. Most of the non-accepted offspring will be rejected and will signal SLiM to go back to step 1 to regenerate child candidates and run all the following steps again. Here, we provided another parameter δ so that the non-accepted offspring have a $\delta/(1 + \delta)$ chance of escaping rejection. This shortcut can help improve computational efficiency especially when the relatedness checking parameter D is very small.

6. Acceptance: When a child is accepted from slot i, SLiM will move on to the next slot $i + 1$; otherwise, SLiM will go back to step 1 to regenerate child candidates for slot i.

By varying the five parameters (G, B, C, D, δ) in the above steps, we can model inbreeding with a wide range of inbreeding levels. Here, we vary the value of parameter δ to allows finer control of inbreeding levels.

Modifying the inbreeding coefficient by adjusting the rate of selfing When a mosquito feeds on a single, monoclonal infection, female and male gametocytes are of the same source, the mating involved in this process resembles a special form of assortative mating, selfing. SLiM provides a simple interface (setSelfingRate) to set the selfing rate of a subpopulation, *i.e.* the fraction of the population that is generated due to selfing. By setting the selfing rate to different values between 0.0 and 1.0, we can obtain different levels of inbreeding.

Supplementary Note 3 Impact of Different Levels and Types of Inbreeding on Selection Bias and Bias Correction

1 Impact of Inbreeding modeled by shrinking population size

Inbreeding modeled via shrinking population size in the multiple-population model We simulated different levels of inbreeding by varying the parameter N_0 from 10,000 to 300 in the multiple-population model. This variation led to a corresponding increase in the inbreeding potential (defined above) from 0.0005 to 0.0101. As inbreeding intensified (Supplementary Figure 11), from row 1 to row 4), we observed a rise in both the baseline IBD sharing and the height of IBD peaks (H), averaged across chromosomes. With lower levels of inbreeding, the effects of positive selection noticeably blurred population structure (see Supplementary Figure 11b, left vs. middle column), but these effects were correctable (Supplementary Figure 11b,

middle vs. right columns). However, at a very low N_0 value (300), despite pronounced IBD peaks, positive selection had a diminished local impact on population structure inference, making correction less vital. The high IBD peaks and reduced selection bias in population structure for high inbreeding (Supplementary Figure 11b bottom row), seemingly inconsistent, are in accordance when we check the quantitative metric – peak impact index average over chromosomes (I). The peak impact index decreases as the inbreeding potential (f) increases, likely due to the background/baseline IBD sharing level increasing to a larger extent than that of the local IBD peaks. This trend suggests that the (local) biases introduced by positive selection in high-inbreeding contexts are less pronounced, potentially negating the need for selection correction. A higher level of discussion on the interplay between local and non-local selection effects is provided at the end of Supplementary Note 3.

Inbreeding modeled via shrinking population size in the single-population model For the single-population model, we varied the value of N_0 from 10,000 to 500, resulting in inbreeding potential estimates ranging from 0.0023 to 0.0302, as depicted in Supplementary Figure12a. This model revealed a strong correlation between the baseline inbreeding potential (f) and the impact of positive selection on IBD-based effective population size (N_e) estimates (see Supplementary Figure12b). Specifically, selection resulted in a more pronounced underestimation of N_e in populations with lower inbreeding levels (larger N_0) compared to those with higher inbreeding (smaller N_0). This trend aligns with the negative correlation we found in multiple-population model between the peak impact index (I) and the baseline inbreeding potential (f), suggesting that the extent of inbreeding significantly influences the bias introduced by positive selection in N_e estimations.

2 Impact of Inbreeding modeled by assortative mating

Here, we show varying levels of inbreeding controlled by the δ parameter for both the single and multiple-population models with $N_0 = 1{,}000$. The full set of parameters for each simulation is provided in our GitHub simulation Readme file.

Inbreeding modeled via positive assortative mating in the multiple-population model In this set of simulations, the value of parameter δ varies from 1000 to 0.001, corresponding to inbreeding potentials from 0.0034 to 0.1224. The simulations with $\delta = 1000$ (the first row in Supplementary Figure13a), serving as controls as $\delta/(1+\delta) \approx 100\%$ escape of high-inbreeding mating, resembles the simulation in Supplementary Figure11a with N_0 =1,000 (row 3). The selection bias in population structure inference is largely consistent with inbreeding modeling via population size reduction (Supplementary Figure11). Generally, high inbreeding potential estimates correspond to very low peak impact indices (Supplementary Figure13a, bottom rows) and little bias of population structure inference (Supplementary Figure13b, bottom rows).

Inbreeding modeled via positive assortative mating in the single-population model The values of parameter δ from 1000 to 0.001 correspond to inbreeding potentials from 0.0155 to 0.3299 (Supplementary Figure14a). As the inbreeding modeled here involves non-random mating, populations with strong inbreeding show a deviation of N_e from the parameter true population size (census size) even in neutral simulations (s = 0.0) (Supplementary Figure 14 b first column, row 4-5). Selection bias correction via IBD peak removal is effective in low inbreeding populations and might not be necessary for extremely high inbreeding populations given the reduced peak impact index (I) and small change in N_e after peak removal (Supplementary Figure14 row 5).

3 Impact of Inbreeding modeled by selfing

Inbreeding modeled via selfing in the multiple-population model We simulated *Pf* genomes under the multiple-population model with different selfing rates from 0 to 0.9 and obtained inbreeding potentials from 0.0034 to 0.2397. Under the selfing inbreeding model, the pattern is different from those of the

previous two strategies. Here, we found the peak impact index is positively correlated with inbreeding potential estimates when selfing rate ≤ 0.7 and negatively correlated when selfing rate > 0.7 (Supplementary Figure15a). In the latter scenario, the previously identified association of high peak impact index with larger correctable selection bias does not hold (Supplementary Figure15b). For instance, when the selfing rate = 0.8, the peaks have a high average peak impact index = 0.134 (Supplementary Figure 15a row 6, second column), but the selection bias in population structure inference is not easy to correct (Supplementary Figure15b row 6, column 2-3). This finding is likely due to a synergistic effect of selfing on positive selection which is supported by Supplementary Figure15c, where the (absolute values of) slopes of allele frequency for the favored mutation dramatically increase as inbreeding increases. Additionally, the global impact indices are also extremely large ($I^G > 5$) for selfing rate ≥ 0.8 (Supplementary Figure17, row 3 column 3). Thus, the effect of selfing-induced inbreeding represents a mixed effect of assortative mating and enhanced selection resulting in dominant global impact as the inbreeding level increases. However, with less extreme selfing ($<$ 0.7), the peak impact index is still a reliable predictor of the magnitude of local selection bias in population structure inference.

Inbreeding modeled via selfing population in the single-population model For the single-population model, we varied the selfing rate from 0 to 0.7 (more extreme rates cause too many genomes to be removed due to high relatedness). As we found in the multiple-population model (Supplementary Figure 15c), where inbreeding via selfing leads to an increased slope of allele frequency trajectory, high inbreeding due to selfing is accompanied by strong selection bias. In this case with selfing rate = 0.7, the selection bias is the strongest. Peak impact index corresponds well with the extent of selection bias, with peak impact index being the highest when the selfing rate = 0.7. However, the selection correction is less effective especially with a selfing rate = 0.7, likely due to a strong global impact $I^G = 1.70$ (compared to $I^G = 1.10$ when selfing rate = 0.0, see Supplementary Figure17 and below for details).

4 Local impact and global impact of selective sweeps under varying levels of inbreeding

In summary, we conducted a detailed analysis to quantitatively explore the relationship between inbreeding and the local/global impacts of selective sweeps by summarizing the above simulations. We utilized the peak impact index (I) to assess the local impact and the global impact index (I^G) for the broader, chromosomal impact. Our findings, illustrated in Supplementary Figure 17, show that increased inbreeding tends to diminish the local impact of selective sweeps but correlates with a more pronounced global impact. This pattern holds true in scenarios where inbreeding is simulated either by shrinking population size (Supplementary Figure17 column 1) or through assortative mating (Supplementary Figure17 column 2). However, an interesting divergence occurs when inbreeding is modeled via selfing (Supplementary Figure17 column 3). In these cases, both local and global impacts intensify as inbreeding increases, potentially due to the synergistic effects of selfing and selection, as seen in Supplementary Figure15c. This observation suggests that the interplay between inbreeding and selection is complex and may require further expanded simulation analyses for a comprehensive understanding.

Overall, our results, barring a few outlier cases, support the hypothesis that higher levels of inbreeding are associated with an increased global impact of positive selection. This, in turn, implies that the advantages of selection correction diminish in highly inbred populations. The extent of selection bias and the necessity of bias correction can be quantified with the peak impact index especially in populations with low inbreeding levels.

References

[1] Zhu, S. J., Almagro-Garcia, J. & McVean, G. Deconvolution of multiple infections in Plasmodium falciparum from high throughput sequencing data. *Bioinformatics (Oxford, England)* **34**, 9–15 (2018).

[2] Zhu, S. J. *et al.* The origins and relatedness structure of mixed infections vary with local prevalence of P. Falciparum malaria. *eLife* **8**, e40845 (2019).

[3] Browning, S. R. & Browning, B. L. Accurate Non-parametric Estimation of Recent Effective Population Size from Segments of Identity by Descent. *American Journal of Human Genetics* **97**, 404–418 (2015).

[4] Hijmans, R. J., Guarino, L., Cruz, M. & Rojas, E. Computer tools for spatial analysis of plant genetic resources data: 1. DIVA-GIS. *Plant genetic resources newsletter* 15–19 (2001).

[5] Walsh, B. & Lynch, M. *Evolution and Selection of Quantitative Traits* (Oxford University Press, New York, NY, 2018).

[6] Anderson, T. J. C., Paul, R. E. L., Donnelly, C. A. & Day, K. P. Do malaria parasites mate non-randomly in the mosquito midgut? *Genetics Research* **75**, 285–296 (2000).

[7] Haller, B. C. & Messer, P. W. SLiM 3: Forward Genetic Simulations Beyond the Wright–Fisher Model. *Molecular Biology and Evolution* **36**, 632–637 (2019).

[8] Haller, B. C., Galloway, J., Kelleher, J., Messer, P. W. & Ralph, P. L. Tree-sequence recording in SLiM opens new horizons for forward-time simulation of whole genomes. *Molecular Ecology Resources* **19**, 552–566 (2019).
