# Supplementary material for: Strong positive selection biases identity-by-descent-based inferences of recent demography and population structure in Plasmodium falciparum
Source: Nat Commun. 2024 Mar 20;15:2499. doi: 10.1038/s41467-024-46659-0 (PMC10954658; doi:10.1038/s41467-024-46659-0)
Supplement: Supplementary file 3 — Reporting Summary [file 41467_2024_46659_MOESM3_ESM.pdf]

nature portfolio

Corresponding author(s): Shannon Takala-Harrison

Last updated by author(s): Feb 16, 2024

Reporting Summary

Nature Portfolio wishes to improve the reproducibility of the work that we publish. This form provides structure for consistency and transparency in reporting. For further information on Nature Portfolio policies, see our Editorial Policies and the Editorial Policy Checklist.

Statistics

For all statistical analyses, confirm that the following items are present in the figure legend, table legend, main text, or Methods section.

n/a	Confirmed	
☐	☒	The exact sample size (*n*) for each experimental group/condition, given as a discrete number and unit of measurement
☐	☒	A statement on whether measurements were taken from distinct samples or whether the same sample was measured repeatedly
☐	☒	The statistical test(s) used AND whether they are one- or two-sided<br>*Only common tests should be described solely by name; describe more complex techniques in the Methods section.*
☐	☒	A description of all covariates tested
☐	☒	A description of any assumptions or corrections, such as tests of normality and adjustment for multiple comparisons
☐	☒	A full description of the statistical parameters including central tendency (e.g. means) or other basic estimates (e.g. regression coefficient) AND variation (e.g. standard deviation) or associated estimates of uncertainty (e.g. confidence intervals)
☐	☒	For null hypothesis testing, the test statistic (e.g. *F*, *t*, *r*) with confidence intervals, effect sizes, degrees of freedom and *P* value noted<br>*Give P values as exact values whenever suitable.*
☒	☐	For Bayesian analysis, information on the choice of priors and Markov chain Monte Carlo settings
☐	☒	For hierarchical and complex designs, identification of the appropriate level for tests and full reporting of outcomes
☒	☐	Estimates of effect sizes (e.g. Cohen's *d*, Pearson's *r*), indicating how they were calculated

Our web collection on statistics for biologists contains articles on many of the points above.

Software and code

Policy information about availability of computer code

Data collection	no software was used for data collection.
Data analysis	Source code for custom packages or scripts are all publicly available as GitHub repositories under the MIT license, including: (1) tskibd, the true IBD inference tool (https://github.com /bguo068/tskibd, v0.0.1); (2) ibdutils, a small python package to facilitate identity-by-descentbased analysis (https://github.com/bguo068/ibdutils, v0.1.0); (3) snp call nf: a Nextflow pipeline for Plasmodium SNP calling (https://github.com/bguo068/snp_call_nf, v0.1.0); (4) posseleff simulations: a Nextflow pipeline to assess the impact of positive selection on Identity-byDescent (IBD)-based inferences, utilizing population genetic simulations and true IBD methodologies. (https://github.com/bguo068/posseleff_simulations, v0.1.0); (5) posseleff empirical: a Nextflow pipeline for analyzing empirical WGS data for the effect of positive selection on IBD-based inference (https://github.com/bguo068/posseleff_empirical, v0.1.1).<br><br>Open source software/packages used within the above custom softeare/pipeline and their version numbers are specified within the Conda environment recipe or package descriptions of each GitHub repository.

For manuscripts utilizing custom algorithms or software that are central to the research but not yet described in published literature, software must be made available to editors and reviewers. We strongly encourage code deposition in a community repository (e.g. GitHub). See the Nature Portfolio guidelines for submitting code & software for further information.

Data

Policy information about availability of data

All manuscripts must include a data availability statement. This statement should provide the following information, where applicable:

- Accession codes, unique identifiers, or web links for publicly available datasets
- A description of any restrictions on data availability
- For clinical datasets or third party data, please ensure that the statement adheres to our policy

Reads of new whole genome sequence data (n = 640 Pf isolates) are deposited to NCBI Sequence Read Archive (SRA) and publicly available under the accession number PRJNA1004408 (https://www.ncbi.nlm.nih.gov/bioproject/?term=PRJNA1004408). Other publicly available WGS data can be found in MalariaGEN Catalogue of Genetic Variation in P. falciparum v6.0, Pf 6 (meta information: https://www.malariagen.net/data/catalogue-genetic-variation-p-falciparum-v6.0; raw reads: https://www.ebi.ac.uk/ena/browser/home, n = 2,978 analyzed) and NCBI SRA under the accession number PRJNA312679 (https://www.ncbi.nlm.nih.gov/bioproject/?term=PRJNA312679, n = 111 analyzed). The accession numbers and hyperlinks for each individual isolate are provided in Supplementary Data 1. Source Data for relevant Figures, Supplementary Figures and Supplementary Tables are available in a Source Data file. The sequence of reference genome PlasmoDB-44 Pfalciparum3D7 is available at PlasmoDB (https://plasmodb.org/common/downloads/release-44/Pfalciparum3D7/fasta/data).

Research involving human participants, their data, or biological material

Policy information about studies with human participants or human data. See also policy information about sex, gender (identity/presentation), and sexual orientation and race, ethnicity and racism.

Reporting on sex and gender	This study examines malaria parasite genomic data. Most of the data were publicly available. Newly reported data generated by our group was generated from malaria parasites infecting patients participating in drug trials or malaria surveys conducted in Cambodia and Thailand by the Armed Forces Research Institute of Medical Sciences. Blood samples were collected from patients with malaria without restriction on specific sex. Patient sex was not relevant to our analysis of malaria parasite population genetics, was not used in the analysis, and is not reported with the study data.
Reporting on race, ethnicity, or other socially relevant groupings	Samples were collected from patients residing in Cambodian and Thailand. Patient race/ethnicity was not relevant to the our analysis of malaria parasite population genetics, was not used in the analysis, and is not reported with the study data.
Population characteristics	Study participants were at least 13 years of age and had clinical malaria diagnosed as P. falciparum.
Recruitment	Study participants were recruited from local health centers upon presenting with clinical falciparum malaria. If patients met the inclusion criteria, they underwent the informed consent process. All adult participants provided informed consent. All minors <18 years of age provided assent with a parent/guardian providing consent.
Ethics oversight	Most of the data included in the study are publicly available. In-house P. falciparum WGS data were generated from samples collected from individuals with symptomatic malaria participating in research studies conducted by the Armed Forces Research Institute of Medical Sciences with approval from the Walter Reed Army Institute of Research and local ethics committees. All study participants or their guardians provided informed consent. Parasite sequencing and genomic analyses were undertaken with the approval of the University of Maryland School of Medicine Institutional Review Board.

Note that full information on the approval of the study protocol must also be provided in the manuscript.

Field-specific reporting

Please select the one below that is the best fit for your research. If you are not sure, read the appropriate sections before making your selection.

☒ Life sciences ☐ Behavioural & social sciences ☐ Ecological, evolutionary & environmental sciences

For a reference copy of the document with all sections, see nature.com/documents/nr-reporting-summary-flat.pdf

Life sciences study design

All studies must disclose on these points even when the disclosure is negative.

Sample size	This is a secondary data analysis of existing malaria parasite genomic data. Sample size was determined based on availability of parasite sequence data for a given geographic location.
Data exclusions	Parasite isolates with low quality sequence data were excluded based on criteria detailed in the methods section of the manuscript.
Replication	Empirical analyses included all available parasite WGS data for joint analyses. For some analyses, such as IBD-based Ne estimation and population structure inference, we used chromosome bootstrapping to demonstrate the robustness of the results.<br><br>For simulation studies, analyses were replicated at different levels, such as n = 14 chromosomes for each genome set and n = 30 populations (sets of genomes). The analysis results are concordant with each other across the replications.

Randomization	This study is not a clinical trial and did not require randomization to different interventions or experimental groups.
Blinding	Blinding was not required, as our study did not involve assignment of individuals to different experimental groups.

Reporting for specific materials, systems and methods

We require information from authors about some types of materials, experimental systems and methods used in many studies. Here, indicate whether each material, system or method listed is relevant to your study. If you are not sure if a list item applies to your research, read the appropriate section before selecting a response.

Materials & experimental systems

n/a	Involved in the study	
☒	☐	Antibodies
☒	☐	Eukaryotic cell lines
☒	☐	Palaeontology and archaeology
☒	☐	Animals and other organisms
☒	☐	Clinical data
☒	☐	Dual use research of concern
☒	☐	Plants

Methods

n/a	Involved in the study	
☒	☐	ChIP-seq
☒	☐	Flow cytometry
☒	☐	MRI-based neuroimaging
