# Supplementary material for: Strong positive selection biases identity-by-descent-based inferences of recent demography and population structure in Plasmodium falciparum
Source: Nat Commun. 2024 Mar 20;15:2499. doi: 10.1038/s41467-024-46659-0 (PMC10954658; doi:10.1038/s41467-024-46659-0)
Supplement: Supplementary file 4 — Description of Additional Supplementary Files [file 41467_2024_46659_MOESM4_ESM.pdf]

Description of Additional Supplementary Files

File Name: Supplementary Data 1

Description: An Excel spreadsheet contains the WGS data accession numbers at the per-sample (run) level
